# Dynamic Molecular Evolution of Mammalian Homeobox Genes: Duplication, Loss, Divergence and Gene Conversion Sculpt PRD Class Repertoires

**DOI:** 10.1007/s00239-021-10012-6

**Published:** 2021-06-07

**Authors:** Thomas D. Lewin, Amy H. Royall, Peter W. H. Holland

**Affiliations:** Department of Zoology, University of Oxford, 11a Mansfield Road, Oxford, OX1 3SZ UK

**Keywords:** Etchbox, Genome evolution, Homeodomain, Positive selection, Tandem duplication

## Abstract

The majority of homeobox genes are highly conserved across animals, but the eutherian-specific ETCHbox genes, embryonically expressed and highly divergent duplicates of *CRX*, are a notable exception. Here we compare the ETCHbox genes of 34 mammalian species, uncovering dynamic patterns of gene loss and tandem duplication, including the presence of a large tandem array of *LEUTX* loci in the genome of the European rabbit (*Oryctolagus cuniculus*). Despite extensive gene gain and loss, all sampled species possess at least two ETCHbox genes, suggesting their collective role is indispensable. We find evidence for positive selection and show that *TPRX1* and *TPRX2* have been the subject of repeated gene conversion across the Boreoeutheria, homogenising their sequences and preventing divergence, especially in the homeobox region. Together, these results are consistent with a model where mammalian ETCHbox genes are dynamic in evolution due to functional overlap, yet have collective indispensable roles.

**Supplementary Information:**

The online version contains supplementary material available at 10.1007/s00239-021-10012-6.

## Introduction

Homeobox genes encode a diverse set of transcription factors found across the Eukaryota, each of which has a characteristic DNA-binding homeodomain of around 60 amino acids (Duboule 1994; Derelle et al. 2007; Holland et al. 2007). Many homeobox genes play critical roles in early embryo patterning and cell fate specification (Wellik 2007; Mallo et al. 2010; Holland 2013) and, as fundamental components of developmental gene regulatory networks, are generally highly conserved over large phylogenetic distances (Bürglin and Affolter 2016). Indeed, most research on homeobox genes has focused on highly conserved examples, such as *HOX* (e.g. Burke et al. 1995; Duboule 2007; Maeda and Karch 2009; Mallo et al. 2010), *PAX* (e.g. Gruss and Walther 1992; Dahl et al. 1997; Blake and Ziman 2014), *POU* (e.g. Herr et al. 1988; Phillips and Luisi 2000) and *LIM* (Sheng et al. 1996; Hobert and Westphal 2000; Costello et al. 2015) genes.

In contrast, there are a smaller number of fast-evolving, taxon-specific homeobox genes found in some animals, including genes expressed during nematode (Bürglin and Cassata 2002; Mukherjee and Bürglin 2007), lepidopteran (Chai et al. 2008; Ferguson et al. 2014), spiralian (Paps et al. 2015; Morino et al. 2017) and mammalian (Maeso et al. 2016) embryonic development. We consider these genes to be fast-evolving on the basis of extensive amino acid change over relatively short timescales, following their origin by gene duplication. In some cases, the amino acid divergence from the deduced parental gene is so great as to cloud insights into their origins, unless additional information such as chromosomal location is also used.

Within mammals, the clearest examples of fast-evolving homeobox genes are *NANOGNB*, a member of the ANTP class (Dunwell and Holland 2017), and several loci classified within the PRD class, although they lack a PAIRED box. These genes include *CPHX1* and *CPHX2*, the *RHOX* genes, the double homeobox genes *DUXA* and *DUXB*, and the Eutherian Totipotent Cell Homeobox (ETCHbox) genes (MacLean et al. 2005; Töhönen et al. 2015; Madissoon et al. 2016; Maeso et al. 2016). Six paralogous groups make up the ETCHbox genes—*Arginine-Fifty Homeobox* (*ARGFX*), *Divergent Paired-Related Homeobox* (*DPRX*), *Leucine-Twenty Homeobox* (*LEUTX*), *Parent of ARGFX* (*PARGFX*), *Tetra-Peptide Repeat Homeobox 1* (*TPRX1*) *and Tetra-Peptide Repeat Homeobox 2* (*TPRX2*)*—*all derived by duplication and extensive sequence divergence from the OTX-family member *Cone-rod homeobox* (*CRX*) (Booth and Holland 2007; Maeso et al. 2016). These genes are a synapomorphy of the Eutheria. They are absent from monotremes and marsupials, having arisen in the lineage leading to the eutherians, after which they underwent rapid asymmetric evolution and diverged extensively from *CRX* (Maeso et al. 2016).

The ETCHbox genes are notable for their remarkably specific temporal expression patterns. Though there are slight variations, human and cow ETCHbox genes are expressed between the 4-cell stage and early blastocyst of the preimplantation embryo and then never expressed again (Maeso et al. 2016). Despite being extensively duplicated, mouse ETCHbox genes are also expressed in the preimplantation embryo (Rajkovic et al. 2002; Cheng et al. 2007; Saito et al. 2010; Maeso et al. 2016; Royall et al. 2018). Furthermore, data from ectopic expression experiments in human fibroblasts and human embryonic stem cells suggest that ETCHbox genes regulate preimplantation embryo-expressed genes and that *LEUTX* has a role in embryonic genome activation (Jouhilahti et al. 2016; Maeso et al. 2016).

Maeso et al. (2016) published the most extensive characterisation of ETCHbox gene complements to date, comparing nine eutherian species. These data suggested that the genes are more dynamic than typical homeobox genes, with a high prevalence of gene duplication and loss, contrasting with the genes’ conserved and highly specific expression pattern. However, this analysis was limited by sparse phylogenetic coverage and the low-quality genome assemblies available at the time. Katayama et al. (2018) performed a deeper analysis of *LEUTX* evolution, but this study was restricted to the one gene and without analysis of gene loss. Recent improvements to long-read DNA sequencing mean that many additional mammalian genome sequences are now available, assembled to high contiguity and accuracy. These data give a timely opportunity to describe the number and organisation of ETCHbox genes across the eutherian phylogeny, which is a necessary step towards understanding the reasons underpinning their unusual pattern of evolution.

The causes and consequences of ETCHbox genes’ dynamic evolution are yet to be elucidated. It has been proposed that their dynamism may be driven by a possible role in the evolution of reproductive traits in mammals, such as placentation, which is highly variable between eutherians (Maeso et al. 2016), or due to selection in some lineages for shorter gestation times (Katayama et al. 2018). Alternatively, the genes’ dynamic evolution may be a consequence of partial functional redundancy, which would cause relaxed selection on loss-of-function mutations, or distributed robustness, where the perturbation of one part of a system (loss of a gene) is compensated by non-redundant parts (other genes) (Wagner 2005; Royall et al. 2018). Finally, the genes may lack an important function, and, therefore, their high rates of pseudogenisation and loss would be a consequence of relaxed selection.

Here we analyse publicly available genome sequences to produce a dataset of ETCHbox repertoires for 34 mammals. We focus particularly on assemblies made with long-read DNA sequencing technology and species chosen to optimise phylogenetic coverage, allowing us to deduce with confidence the underlying patterns and pathways of gene duplication and loss. We uncover large arrays of tandem ETCHbox duplicates across multiple species and show that the ETCHbox genes have been the subject of positive selection and concerted evolution.

## Materials and Methods

### Comparative Genomics

Genome sequences for 32 eutherian species were downloaded from the NCBI webpage, focussing on taxa with high contiguity genome assemblies (Online Resource Table S1); this includes re-analysis of species previously analysed using lower quality genome data (Maeso et al. 2016). When possible, genomes sequenced using long-read technologies were utilised, as such data facilitates assessment of tandem gene duplication and gene loss. To include species from every order of the Boreoeutheria (Laurasiatheria and Euarchontoglires), three taxa were included despite lacking long-read assemblies: *Galeopterus variegatus* (Sunda flying lemur, Dermoptera), *Condylura cristata* (star-nosed mole, Eulipotyphla) and *Manis javanica* (Sunda pangolin, Pholidota). High-quality human and mouse genome assemblies were analysed by Maeso et al. (2016) and Royall et al. (2018), respectively, and are used but not recharacterised here, giving a total dataset of 34 species.

*Homo sapiens* (human; Maeso et al. 2016) and *Bos taurus* (cattle; this work) ETCHbox gene structures were verified using transcriptome data. Briefly, for *B. taurus*, RNA-seq reads (Online Resource Table S2; Graf et al. 2014; Jiang et al. 2014; Bernardo et al. 2018; Liu et al. 2018) were obtained from the NCBI Sequence Read Archive (SRA), aligned to the *B. taurus* reference genome ARS-UCD1.2 using STAR version 2.7.0 (Dobin et al. 2013) and assembled into transcripts using StringTie version 1.3.6 (Pertea et al. 2015). Genes of interest were identified in each transcriptome using the Basic Local Alignment Search Tool (BLAST) (Altschul et al. 1990, 1997), and intron/exon boundaries refined by inspection of raw reads using the Integrative Genomics Viewer (IGV) (Robinson et al. 2011).

For species lacking appropriate transcriptome data, ETCHbox genes were identified and annotated using sequence similarity searches of genome assemblies (blastn, blastp, megablast and tblastn; Altschul et al. 1990, 1997; Zhang et al. 2000). Gene identities were assigned using a combination of reciprocal BLAST, neighbouring genes and phylogenetic analysis (MrBayes; Huelsenbeck and Ronquist 2001; Ronquist et al. 2012). Intron/exon boundaries were refined manually using (a) retrogene sequences, (b) comparison to human and cow sequences validated by transcriptome data, and (c) mammalian consensus splice sites (Burset et al. 2000). The first coding exon of ETCHbox genes is very short and highly variable, and was therefore not always identified. Genes were considered probable pseudogenes when there were stop codons, splice site mutations or frameshifts upstream of (or within) the homeobox. Genes with frameshifts or premature stop codons immediately downstream of the homeobox are of unknown functional status. If no gene was identified by BLAST and the expected syntenic region surrounding the gene was split over two or more scaffolds we do not conclude certain gene loss.

For phylogenetic analysis, amino acid sequence alignments were made using Clustal Omega in Seaview version 4.7 (Gouy et al. 2010; Sievers et al. 2011) and phylogenies inferred using MrBayes version 3.2.7a (Huelsenbeck and Ronquist 2001; Ronquist et al. 2012) and rendered using iTOL (Letunic and Bork 2019).

### Estimating Gene Gain and Loss

Two methods were used to assess gene gain and loss. First, ETCHbox genes were grouped into gene families and the stochastic birth and death model in CAFE (De Bie et al. 2006; Han et al. 2013) used to calculate maximum likelihood values of λ and μ (rates of gain or loss, respectively, per gene per million years) and estimate gene numbers at internal nodes. Second, the event-inference parsimony algorithm in Notung version 2.9 (Chen et al. 2000; Durand et al. 2006) was used. Gene trees were generated for each ETCHbox gene as above and Notung run with a duplication-loss model and default parameters (weights: duplications = 1.5, co-divergences = 0.0, losses = 1.0) to reconcile gene and species trees and estimate the timing and minimum weighted number of independent duplication and loss events. To prevent weakly supported branches causing overestimation of gene turnover, gene trees were rearranged using the ‘Rearrange’ function, allowing branches with posterior probabilities < 95% to be reconfigured to minimise duplications and losses. The species tree used was generated using TimeTree (Kumar et al. 2017).

CAFE was also used to test for an acceleration in the rate of gene duplication of each ETCHbox gene compared to other homeobox genes present in mammals using the Monte Carlo sampling procedure described previously (Hahn et al. 2005, 2007). The Viterbi assignment method (De Bie et al. 2006) was used to establish which branches contributed to such accelerations. For the purpose of gene duplication analyses, Cetartiodactyla *TPRX3* genes were assigned as *TPRX2* duplicates, as by Maeso et al. (2016).

### Detecting Gene Conversion

We defined *TPRX1* and *TPRX2* using neighbouring genes and orientation, not sequence: *TPRX1* is upstream of *CRX* and in inverse orientation, *TPRX2* is downstream of *CRX* on the same strand. To test for interlocus gene conversion between *TPRX1* and *TPRX2*, four methods were used, following the guidelines of Mansai and Innan (2010). First, the expected *TPRX* duplication history was compared to Bayesian gene trees to search for phylogenetic incompatibilities. Protein sequence alignments of TPRX1 and TPRX2 were trimmed using Gblocks version 0.91b (Talavera and Castresana 2007), converted to codon alignments using PAL2NAL (Suyama et al. 2006) and compared using the phylogenetic methods outlined above. Second, sequence similarity was assessed by running Biostrings version 2.57.1 (Pagès et al. 2020) in R version 4.0.0 ‘Arbor Day’ (R Core Team 2020) to compute all versus all Needleman-Wunsch (Needleman and Wunsch 1970) global pairwise alignments. Percent nucleotide identities were calculated and plotted using gplots version 3.0.3 (Warnes et al. 2020). To understand whether sequence similarity is constant across the length of the genes, a sliding window analysis was performed using Spider version 1.5.0 (Brown et al. 2012), measuring Kimura 2-parameter (K2P) distance (Kimura 1980) between the *TPRX1* and *TPRX2* genes of a given species in 50 base pair (bp) overlapping windows with increments of 1 bp. Only species with at least one putatively functional copy of both *TPRX1* and *TPRX2* were used.

Third, we tested for incompatibilities between phylogenies built using different partitions of the genes. The HyPhy (Kosakovsky Pond et al. 2005, 2020) programme GARD (Kosakovsky Pond et al. 2006a, b) was run using Datamonkey (Weaver et al. 2018) with default parameters on codon alignments of all *TPRX1* and *TPRX2* genes (GARD was also run on alignments of *Oryctolagus cuniculus* and *Microcebus murinus LEUTX* tandem duplicates and *Peromyscus leucopus TPRX* and *LEUTX* genes)*.* GARD uses an aggressive population-based hill climber to search multiple sequence alignments for phylogenetic incongruity and identify putative gene conversion and recombination breakpoints. The AIC_C_ (small sample Akaike Information Criterion) was used to select the model with the best fit to the data, with Akaike weights (w_i_) calculated using the R package qpcR version 1.4.1 and used to assist model selection (Akaike 1974; Sugiura 1978; Hurvich and Tsai 1989; Burnham and Anderson 2002; Wagenmakers and Farrell 2004; Ritz and Spiess 2008). The alignment was split into two partitions based on the breakpoint identified by GARD, and Bayesian phylogenies of each partition built as above. To measure tree dissimilarity, the tqDist algorithm (Sand et al. 2014) was implemented in the R package Quartet version 1.2.0 (Smith 2020) to calculate quartet distance (Estabrook et al. 1985) and quartet divergence (Smith 2019). Finally, GENECONV version 1.81a (Sawyer 1989) was used to identify putative gene conversion events by searching for fragments of sequences with sufficient nucleotide similarity to suggest gene conversion. GENECONV was run with default parameters apart from /lp (implements pairwise comparisons), /w123 (creates reproducible results by initiating at the same random seed number) and -gscale = 2 (allows mismatches in the conversion tracts with a penalty of 2). GENECONV returns *p* values based on 10,000 permutations for fragments found with global (corrected for multiple comparisons) and pairwise sequence comparisons (corrected for alignment length but not multiple sequence comparisons). Given a significance threshold of 0.05, it is expected that if there was no gene conversion in the dataset, then 69 of the 1378 pairwise comparisons would produce false positives. We identified 613 events, suggesting that the majority are not false positives. Furthermore, as a negative control, GENECONV was run with option -Randomize_sites; this permutes sites once and therefore removes any gene conversion signal. This identified just seven gene conversion events, again suggesting that the events detected above are not false positives. Events identified in the negative control analysis were discarded from the results. Only putatively functional genes were included in the gene conversion analysis, and *Mus musculus* and *Peromyscus leucopus Obox* (*TPRX2*) genes were also omitted because they show extreme lineage-specific sequence divergence and their inclusion may disrupt analysis.

### Tests for Accelerated Divergence and Positive Selection

To test for changes in the rate of homeodomain sequence evolution, MEGA X (Kumar et al. 2018; Stecher et al. 2020) was used to undertake Tajima’s relative rate test (Tajima 1993) (*α* = 0.05). Each ETCHbox homeodomain was compared to its conspecific CRX protein, using a marsupial CRX sequence (*Monodelphis domestica*) as an outgroup. Where there are lineage-specific duplications, only one duplicate was used. The Benjamini-Yekutieli (Benjamini and Yekutieli 2001) false discovery rate method (false discovery rate = 0.05) was used to correct for multiple testing as it does not require independence of tests.

Episodic positive selection in ETCHbox genes was detected using the HyPhy (Kosakovsky Pond et al. 2005, 2020) Branch-Site Unrestricted Statistical Test for Episodic Diversification (BUSTED) (Murrell et al. 2015) via Datamonkey (Weaver et al. 2018) with default parameters using codon alignments generated with PAL2NAL (Suyama et al. 2006) and phylogenies reflecting known species relationships. To test for pervasive positive selection, pamlX (Xu and Yang 2013) was used to run CODEML (Model = 0, NSsites = 0, 1, 2, 7, 8) in Phylogenetic Analysis by Maximum Likelihood (PAML) version 4.8 (Yang 1997, 2007). Sites with a gap in more than 50% of sequences were removed, and CODEML run with the option cleanData = 0. Likelihood ratio tests (LRTs) were used to compare model 2 (M2, positive selection model) to model 1 (M1, nearly neutral model) and model 8 (M8, beta and ω model—positive selection) to model 7 (M7, beta model—no positive selection).

The HyPhy Mixed Effects Model of Evolution (MEME) (Murrell et al. 2012), which uses mixed-effects branch-site models, was used to detect specific codon sites evolving under episodic positive selection. MEME is preferred to the branch-site mode of CODEML because it does not require a priori specification of branches to be tested but retains good statistical power (Lu and Guindon 2014). Sites with a gap in more than 50% of sequences were removed from this analysis. Position of residues in relation to homeodomain structure was deduced by comparative structural modelling to the PRD-class homeodomain of *Drosophila melanogaster* Aristaless (Al) in complex with DNA (RCSB Protein Data Bank entry 3LNQ; Berman et al. 2000; Miyazono et al. 2010) using Modeller (Šali and Blundell 1993) implemented in UCSF Chimera 1.15 (Pettersen et al. 2004).

RELAX (Wertheim et al. 2015) was run with default parameters using codon alignments of ETCHbox and *CRX* homeoboxes to test for relaxed selection in each ETCHbox gene versus a reference group of six *CRX* genes (*Canis lupus familiaris*, *Condylura cristata*, *Equus caballus*, *Homo sapiens*, *Mus musculus* and *Ovis aries*).

*Mus musculus* and *Peromyscus leucopus Obox* (*TPRX2*) genes and genes with frameshifts or early stop codons downstream of the homeodomain were omitted from the selection analysis. Furthermore, the phylogenetic incongruity caused by gene conversion could lead to inaccurate results when testing for selection (Anisimova et al. 2003; Shriner et al. 2003; Kosakovsky Pond et al. 2006b). To account for gene conversion in the *TPRX* genes, we used the gene conversion breakpoint identified by GARD (Kosakovsky Pond et al. 2006a, b) to partition the alignment into two sections. MrBayes (Huelsenbeck and Ronquist 2001; Ronquist et al. 2012) was used as above to calculate gene trees for each partition. The above methods were then performed separately for each of the two partitions.

## Results

### Identification of ETCHbox Genes in Eutherian Genomes

**Fig. 1:**
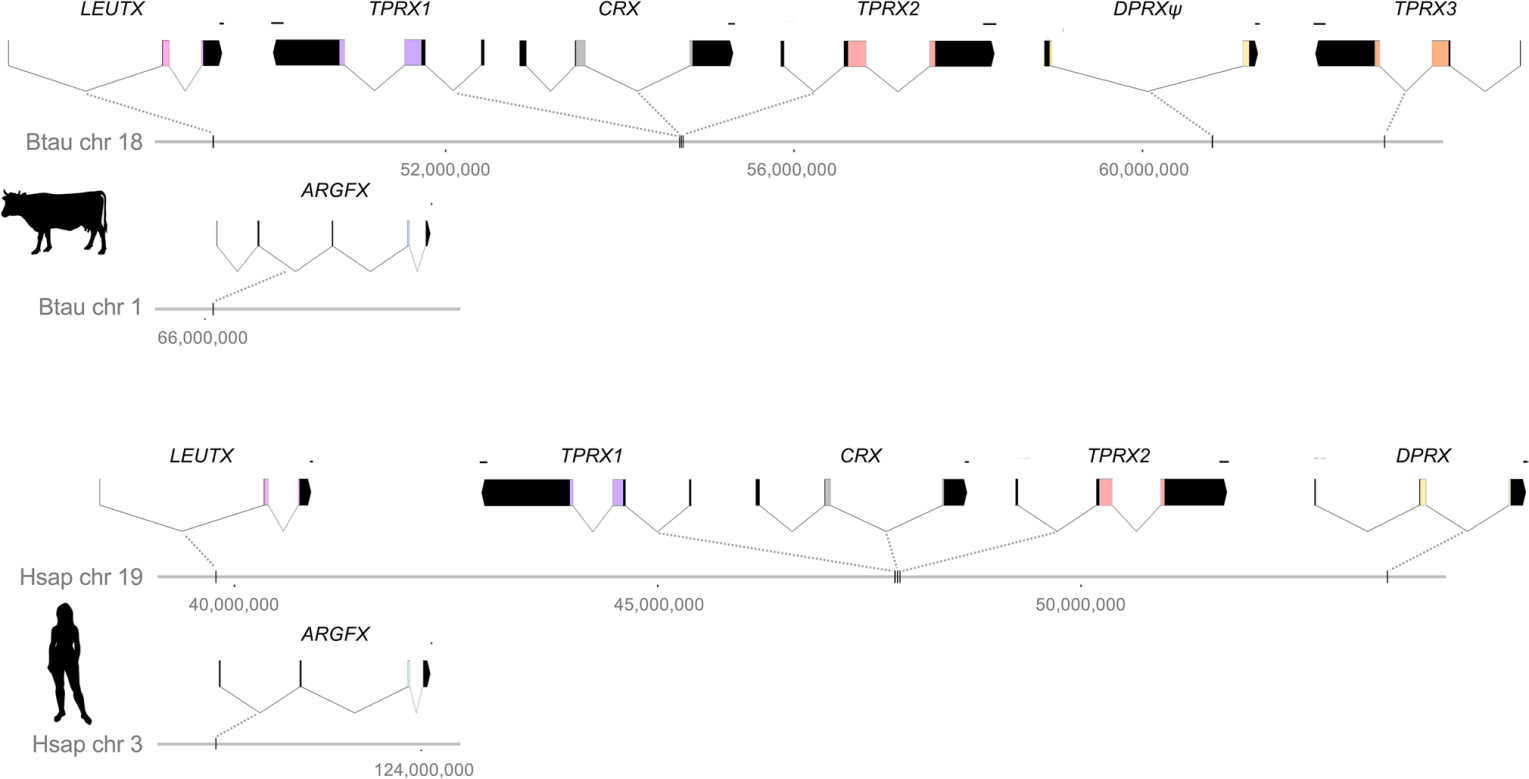
ETCHbox repertoires of *Homo sapiens* (humans) and *Bos taurus* (cattle), with gene structures as determined using transcriptome assemblies. Horizontal grey bars represent chromosomes, vertical black bars represent the genomic position of ETCHbox genes. For gene structure representations, coding regions are shown in black, homeoboxes in colour. Untranslated regions (UTRs) are not shown. Black scale bars at 3’ end of genes = 100 bp. *DPRX*, *LEUTX*, *TPRX1*, *TPRX2* (and *B. taurus* *TPRX3*) form a loose cluster on a single chromosome (*B. taurus* chromosome 18, *H. sapiens* chromosome 19); *ARGFX* has translocated to another chromosome (*B. taurus* chromosome 1, *H. sapiens* chromosome 3). *TPRX1* and *TPRX2* are located either side of the ETCHbox ‘ancestor’ *CRX*

We first characterised the ETCHbox genes of *Bos taurus* (cattle) using transcriptome data (Fig. 1). *B. taurus* possesses putatively functional *ARGFX*, *LEUTX*, *TPRX1* and *TPRX2* genes, but *DPRX* is a putative pseudogene due to a 2 bp insertion in the homeobox and the loss of exon 1; *PARGFX* has been lost. *B. taurus* also possesses a *TPRX* duplicate, which we refer to as *TPRX3*. Compared with *Homo sapiens* (human), *B. taurus ARGFX* has an additional 5’ coding exon, which extends the reading frame. All genes are located in the same syntenic position as in humans, with *LEUTX*, *TPRX1*, *TPRX2* and *DPRX* (and *TPRX3*) in a loose cluster on chromosome 18 (human chromosome 19), and *ARGFX* separate from the cluster on chromosome 1 (human chromosome 3).

**Fig. 2:**
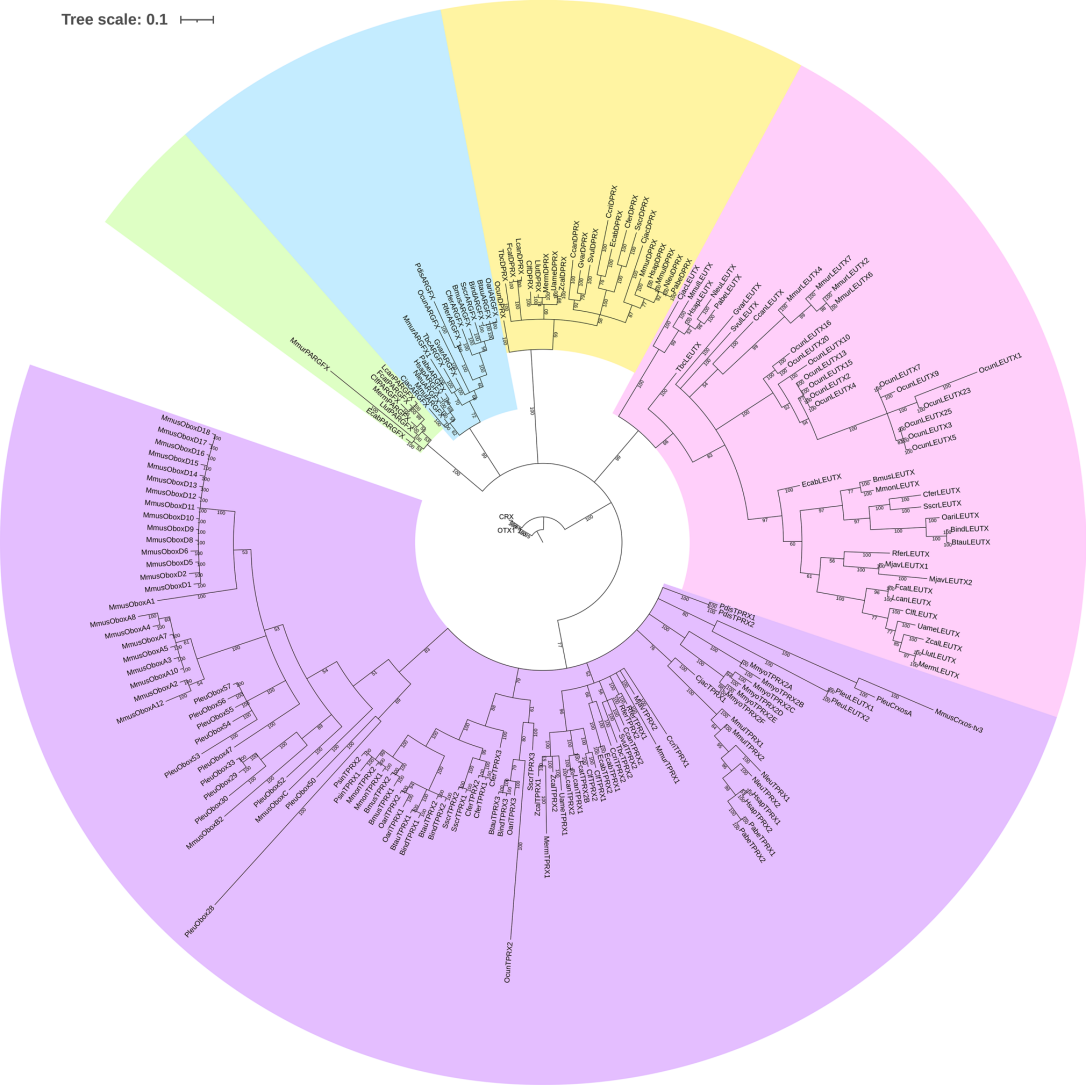
Bayesian gene tree of putatively functional ETCHbox genes identified in this work. Colours highlight ETCHbox gene families; labels show posterior probabilities. The *ARGFX*, *DPRX*, *LEUTX* and *PARGFX* clades are supported by ≥ 99% probabilities. Due to the limited length of the homeodomain (60 amino acids), gene phylogenies do not always recapitulate known relationships between species. *TPRX* duplicates in Cetartiodactyla are referred to as *TPRX3*. The *TPRX1* and *TPRX2* genes of *Mus musculus* and *Peromyscus leucopus* are referred to as *Crxos* and *Obox*, respectively, reflecting their extensive sequence change compared to the ancestral *TPRX* genes. Abbreviations: Bind = *Bos indicus*, Bmus = *Balaenoptera musculus*, Btau = *Bos taurus*, Ccan = *Castor canadensis*, Ccri = *Condylura cristata*, Cfer = *Camelus ferus*, Cjac = *Callithrix jacchus*, Clf = *Canis lupus familiaris*, Ecab = *Equus caballus*, Fcat = *Felis catus*, Gvar = *Galeopterus variegatus*, Hsap = *Homo sapiens*, Lcan = *Lynx canadensis*, Llut = *Lutra lutra*, Mjav = *Manis javanica*, Merm = *Mustela erminea*, Mmon = *Monodon monoceros*, Mmul = *Macaca mulatta*, Mmur = *Microcebus murinus*, Mmus = *Mus musculus*, Mmyo = *Myotis myotis*, Nleu = *Nomascus leucogenys*, Oari = *Ovis aries*, Ocun = *Oryctolagus cuniculus*, Pabe = *Pongo abelii*, Pdis = *Phyllostomus discolor*, Pleu = *Peromyscus leucopus*, Psin = *Phocoena sinus*, Rfer = *Rhinolophus ferrumequinum*, Sscr = *Sus scrofa*, Svul = *Sciurus vulgaris*, Tbc = *Tupaia belangeri chinensis*, Uame = *Ursus americanus*, Zcal = *Zalophus californianus*

**Fig. 3:**
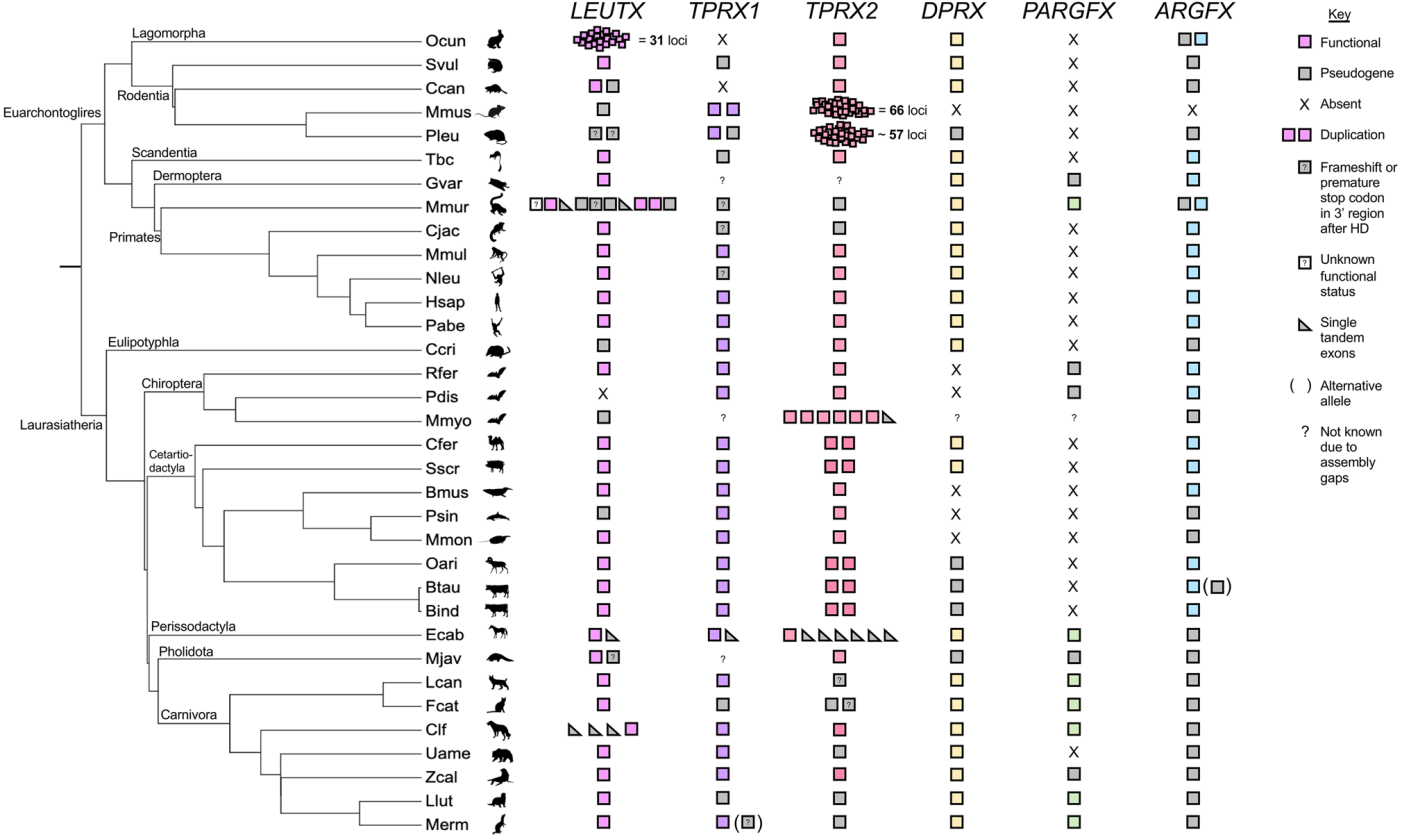
ETCHbox gene repertoires of 34 eutherian mammals. Phylogenetic relationships are based on TimeTree (Kumar et al. 2017). Coloured boxes = putatively functional gene. Multiple boxes = gene duplicates. Black X = no gene remnants (complete gene loss). Grey boxes = putative pseudogene; grey boxes with a black question mark = complete homeodomain but subsequent frameshift or premature stop codon. White boxes with a question mark = unclear functional status due to incomplete assembly in the region. Grey triangles = tandem single exons. Brackets = polymorphism; question marks = assembly gap such that gene presence or absence cannot be determined. HD = homeodomain. Species abbreviations as in Fig. 2

We then characterised the ETCHbox gene repertoires in the genomes of 31 further eutherian species, using a combination of phylogenetics, synteny and reciprocal BLAST searches to assign gene identities (Fig. 2, 3; Online Resource File 1). These methods concurred in almost all cases except for *TPRX1* and *TPRX2* genes, where sequence-based methods disagreed with genomic position; for these genes we use genomic position to assign gene name and assess below whether incongruence is due to gene conversion. The only other discordance occurs in *Peromyscus leucopus* (white-footed mouse), where the two genes at the *LEUTX* locus cluster with rodent *TPRX1* genes, although we find no evidence of gene conversion in this case (GENECONV analysis, no gene conversion fragments identified). In phylogenetic analyses, branch lengths are longer for ETCHbox genes than for their paralogues *CRX* and *OTX1*, indicating a higher amino acid substitution rate. Particularly long branches are observed for *Oryctolagus cuniculus* (European rabbit) *TPRX2* and *LEUTX1*, *Microcebus murinus* (gray mouse lemur) *PARGFX*, and *Mus musculus* (house mouse) and *P. leucopus TPRX1* (= *Crxos*) and *TPRX2* (= *Obox*). Loci with a stop codon, frameshift or splice site disruption in, or upstream from, the homeodomain are inferred to be pseudogenes. The ETCHbox genes frequently spawn retrocopies; these were also characterised, with every sampled species possessing at least one ETCHbox retrogene (Online Resource Table S3a); retrocopies are not clustered, and are found dispersed around the genome (e.g. *Homo sapiens* and *Bos taurus;* Online Resource Table S3b).

The ETCHbox gene repertoires are highly variable between species, with additional tandem gene duplication, pseudogenisation and gene loss occurring repeatedly across eutherians (Fig. 3). Previous work showed that all six ETCHbox genes were present in the ancestor of the Boreoeutheria (Maeso et al. 2016) so absence at a terminal node implies gene loss. All sampled species have lost at least one ETCHbox gene, and each gene has been lost in at least one sampled species. Some gene losses are inferred to have occurred in the ancestors of large clades (e.g. *ARGFX* in the Carnivora and *DPRX* in the Cetruminantia); many other losses are more recent (e.g*. LEUTX* is lost in *Phocoena sinus* [vaquita] but present in other sampled Cetacea species).

In *Mi. murinus*, we note the first putatively functional *PARGFX* gene reported for any member of the Euarchontoglires. *Mi. murinus PARGFX* is in the expected syntenic position and groups phylogenetically with other *PARGFX* genes, albeit on a long branch (Fig. 2). *Galeopterus variegatus* (Sunda flying lemur) also has a detectable *PARGFX* locus, but it is inferred to be a pseudogene.

### Giant Arrays of ETCHbox Genes

We identify several arrays of tandem ETCHbox duplicates, one of the largest of which is an array of *LEUTX* loci in *O. cuniculus*. Previous work detected six loci (Katayama et al. 2018), whereas we detect 27 gene copies in the assembly analysed, of which 14 are putatively functional, 11 are putative pseudogenes and two are of uncertain functional status (Online Resource Fig. S1). We also find four single exons in the cluster, giving a total of 31 loci. This is the largest *LEUTX* expansion discovered and one of the largest ETCHbox expansions, smaller than only those of *Mu. musculus* and *P. leucopus Obox* (*TPRX2*) genes (Royall et al. 2018). An inversion on *O. cuniculus* chromosome 5 has split the array in two, with *LEUTX1* to *LEUTX5* approximately 9 Mb from *LEUTX6* to *LEUTX27*. *Mi. murinus* also has a tandem *LEUTX* expansion of 10 loci, at least three of which are putatively functional, and *Mi. murinus* and *O. cuniculus* are both also notable because they have an *ARGFX* duplication. We find several cases of tandem duplication of single exons, including at *Equus caballus* (domestic horse) *LEUTX*, *TPRX1* and *TPRX2* loci.

It was shown previously that *Mu. musculus* has lost *ARGFX*, *DPRX*, *LEUTX* and *PARGFX* and possesses two *TPRX1* copies (called *Crxos*) and 66 *TPRX2* loci (called *Obox*), all of which are highly divergent in sequence (Maeso et al. 2016; Royall et al. 2018). We asked when the transition to this highly derived state occurred. Our results indicate that this evolved within the rodents. *Sciurus vulgaris* (red squirrel, Sciuridae) and *Castor canadensis* (American beaver, Castoridae) possess putatively functional *DPRX* and *LEUTX* genes, and neither have *TPRX1* or *TPRX2* duplicates (Fig. 3). However, *Peromyscus leucopus* (white-footed mouse, Cricetidae) has two *TPRX1* loci, and no functional *ARGFX*, *DPRX* or *PARGFX*, as in *Mu. musculus.* Furthermore, we detect 57 *P. leucopus TPRX2 (Obox)* loci, of which 12 are putatively functional. Seven of these loci have escaped the *TPRX2* cluster on chromosome 1 and form a separate cluster on chromosome 12. The observation that *P. leucopus TPRX1* and *TPRX2* genes group phylogenetically with *Mu. musculus Crxos* and *Obox*, respectively (Fig. 2), combined with the Notung result that the *TPRX1* duplication and several of the *TPRX2* duplications occurred before the split of *Mu. musculus* and *P. leucopus* (below), suggests that the transition from *TPRX1* and *TPRX2* to *Crxos* and *Obox*-like states occurred before the split of the Muridae and Cricetidae.

### Rates of Gene Duplication

We compared rates of gene duplication and loss for each gene by modelling a stochastic birth–death process using CAFE (De Bie et al. 2006; Han et al. 2013), giving maximum likelihood estimates for the rates of ETCHbox gene gain and loss (events per million years; λ and μ, respectively; Table 1). CAFE was also used to infer likely ancestral gene numbers (Online Resource Fig. S2). Rates of gain (λ) and loss (μ) are highly variable between ETCHbox families. *TPRX2* is the gene most prone to duplication (λ = 0.016) and *PARGFX* most prone to gene loss (μ = 0.019). *ARGFX*, *DPRX* and *PARGFX* have very low rates of gene gain but relatively high rates of loss.

**Table 1:** Duplication and loss in the ETCHbox genes

Gene	λ (gains per gene per million years, CAFE)	μ (losses per gene per million years, CAFE)	Estimated number of gene duplication events (Notung)	Estimated number of gene loss events (Notung)	Number of species with at least one putatively functional gene
*ARGFX*	3.37E-10	6.70E-03	0	5	17
*DPRX*	4.26E-11	3.42E-03	1	8	22
*LEUTX*	8.88E-03	3.32E-03	18	4	29
*PARGFX*	5.84E-11	1.93E-02	0	10	7
*TPRX1*	8.49E-04	6.15E-03	1	10	25
*TPRX2*	1.61E-02	6.33E-03	41	7	28

Probability of duplication or loss (λ and μ) for each ETCHbox gene, estimated by CAFE’s stochastic birth and death model (De Bie et al. 2006; Han et al. 2013), together with estimates of numbers of gene duplication and gene loss events, calculated by Notung (Chen et al. 2000; Durand et al. 2006). Pseudogenes are excluded as duplication events

We find evidence that *LEUTX* (*p* = 0.003) and *TPRX2* (*p* = 0.000) duplicate faster than other homeobox genes. The Viterbi assignment method (De Bie et al. 2006) reveals that the high overall duplication rate of *LEUTX* is primarily a result of changes along the *O. cuniculus* (*p* = 1.503 × 10^–8^) and *Mi. murinus* (*p* = 0.028) branches; the high duplication rate of *TPRX2* is influenced largely by the branches leading to Cetartiodactyla (*p* = 0.027), *Myotis myotis* (greater mouse-eared bat, *p* = 0.001), Muroidea (*p* = 2.774 × 10^–10^), *Mu. musculus* (*p* = 1.774 × 10^–36^) and *P. leucopus* (*p* = 0.003).

A high duplication rate for *LEUTX* and *TPRX2* was also supported by analysis incorporating gene trees, implemented using Notung (Chen et al. 2000; Durand et al. 2006) to estimate the number of duplication and loss events and infer their timings (Table 1 and Online Resource Fig. S3). Gene loss is expected to have most functional relevance when a single copy gene transitions to total absence of a functional gene; we find this occurred most for *PARGFX,* in accordance with CAFE results. There are two cases of apparent gene turnover overestimation: Notung reports the *Camelus ferus* (Bactrian camel) *TPRX3* duplication as independent of other Cetartiodactyla *TPRX3* duplicates, and a *DPRX* duplication at the base of the Caniformia followed by multiple losses. These are likely artefacts caused by the rapidly evolving nature of ETCHbox sequences but do not distort the overall inferences from the analysis.

### Polymorphism in ETCHbox Genes

We find two cases of ETCHbox intraspecific polymorphism where one allele has a frameshift mutation. In *Mustela erminea* (stoat), we identify a putatively functional *TPRX1* in one haplotype of the phased genome assembly while the alternate haplotype has a ‘CC’ dinucleotide insertion causing a frameshift in exon 3. In the *B. taurus* reference genome (ARS-UCD1.2), we find a 13 bp deletion in *ARGFX* exon 2 that causes a frameshift and a premature stop codon before the homeobox, making it a putative pseudogene. We do not find this deletion in several other *B. taurus* datasets (Online Resource Table S2) or in the genome of other Bovidae species (Online Resource Table S4).

### TPRX1 and TPRX2 have been Subject to Repeated Gene Conversion

Interlocus gene conversion is a naturally occurring ‘copy and paste’ process that can take place during double-strand break repair, where DNA sequence from one locus is used to replace DNA sequence at a different locus in the same genome (Chen et al. 2007). The incongruence between gene identity inferred from phylogenetics versus gene position for *TPRX1* and *TPRX2* suggests that gene conversion may have occurred between these loci, as suggested previously (Maeso et al. 2016). However, this hypothesis needs further testing, and it is currently unclear whether the complete loci were affected, when it occurred or how often it occurred in evolution.

**Fig. 4:**
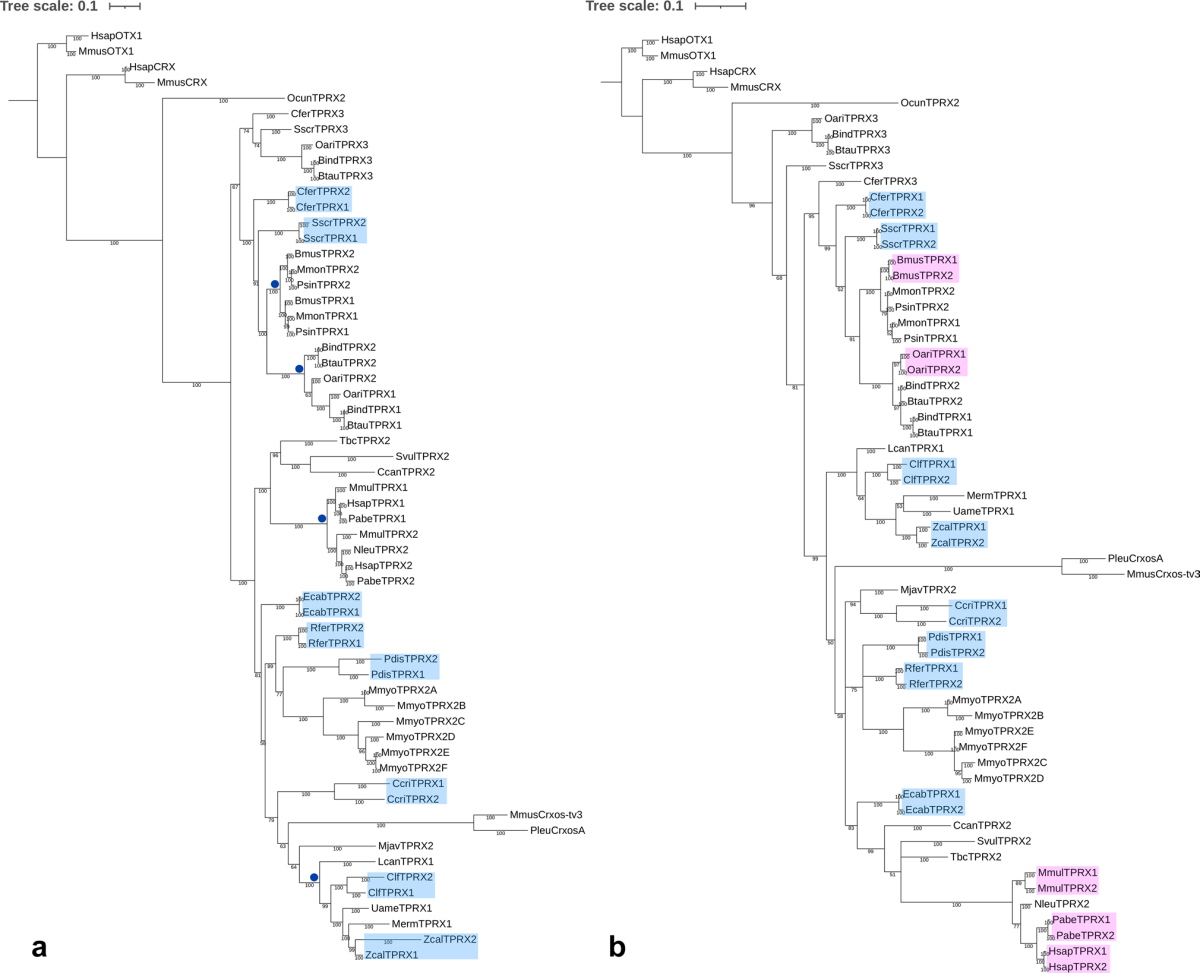
Bayesian phylogenies of putatively functional *TPRX1*, *TPRX2* and *TPRX3* full gene sequences (**a**) and homeoboxes (**b**). Blue boxes highlight cases where conspecific *TPRX1* and *TPRX2* pairs are more closely related to each other than to other sequences. Pink boxes highlight cases that appear on tree b but not tree a. Blue dots mark putative gene conversion events that occurred deeper in the phylogeny. Putative pseudogenes were excluded. Labels show posterior probabilities. Species abbreviations as in Fig. 2

We first investigated these questions using a phylogenetic approach, searching for incompatibilities between the known species tree and the inferred gene tree. Under the null hypothesis of no gene conversion, *TPRX1* and *TPRX2* genes would form separate clades diverging since the base of eutherians; gene conversion would result in paralogues grouping more closely together. Bayesian nucleotide phylogenies of putatively functional *TPRX* genes reveal eight cases where the *TPRX1* and *TPRX2* genes from a given species group together as pairs of sister sequences, suggesting recent gene conversion events in these lineages (Fig. 4a, blue boxes). There are also indications of further gene conversion events deeper in the phylogeny, notably in the stem lineages of Cetacea, Bovidae, Carnivora and Primates (Fig. 4a, blue dots). Intriguingly, we found evidence for additional gene conversion events when phylogenetic analysis was restricted to the homeobox sequence only. This revealed 13 recent conversion events between *TPRX* loci, with five new cases identified in addition to the eight above (Fig. 4b, pink boxes). Several of the additional events are nested within the clades that showed evidence of older gene conversion (Primates, Cetacea), suggesting successive gene conversion events in evolution. The occurrence of successive gene conversion events is also supported by analysis of pairwise nucleotide identity (Online Resource Fig. S4) which, for example, suggests gene conversion at the base of the Cetartiodactyla, then further events within the Bovidae, Cetacea, *Sus scrofa* (domestic pig) and *C. ferus*.

**Fig. 5:**
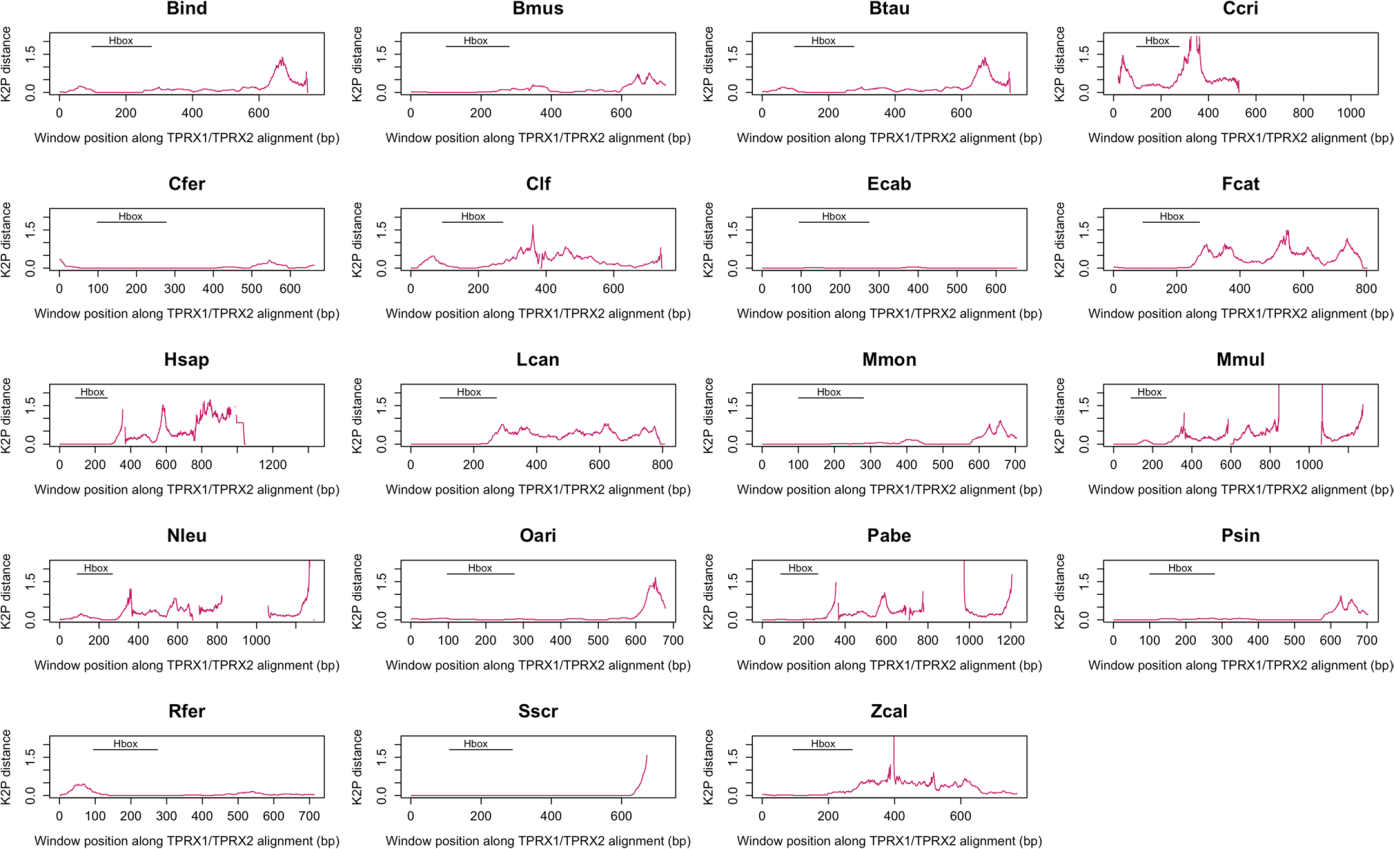
Sequence similarity between *TPRX1* and *TPRX2* genes within a species. Plots show the Kimura 2-parameter (K2P) distance in 50 bp sliding windows between conspecific *TPRX1* and *TPRX2* genes. Higher K2P values indicate more divergent sequences. Gaps in the trace indicate indels in the alignment. The black bar marked ‘Hbox’ demarcates the position of the homeobox in each alignment. For many species, the K2P values increase towards the 3’ end of the gene, suggesting that the *TPRX* genes have been homogenised by gene conversion less at their 3’ ends. Putative pseudogenes were excluded. Species abbreviations as in Fig. 2

Since a homeobox-only tree suggests additional episodes of gene conversion, we hypothesised that the 5’ region of *TPRX* genes is more prone to gene conversion than the 3’ region. To test this, we conducted a sliding window analysis calculating pairwise Kimura 2-parameter (K2P) distances (Kimura 1980) between *TPRX1* and *TPRX2* sequences (Fig. 5). In *Bos indicus* (Zebu cattle), *B. taurus*, *Balaenoptera musculus* (blue whale), *C. ferus*, *Felis catus* (domestic cat), *H. sapiens*, *Lynx canadensis* (Canada lynx), *Monodon monoceros* (narwhal), *Nomascus leucogenys* (northern white-cheeked gibbon), *Ovis aries* (sheep), *P. sinus* and *S. scrofa*, the lowest sequence distances (highest similarities) are at the 5’ end, suggesting this region is more prone to homogenisation via gene conversion.

**Fig. 6:**
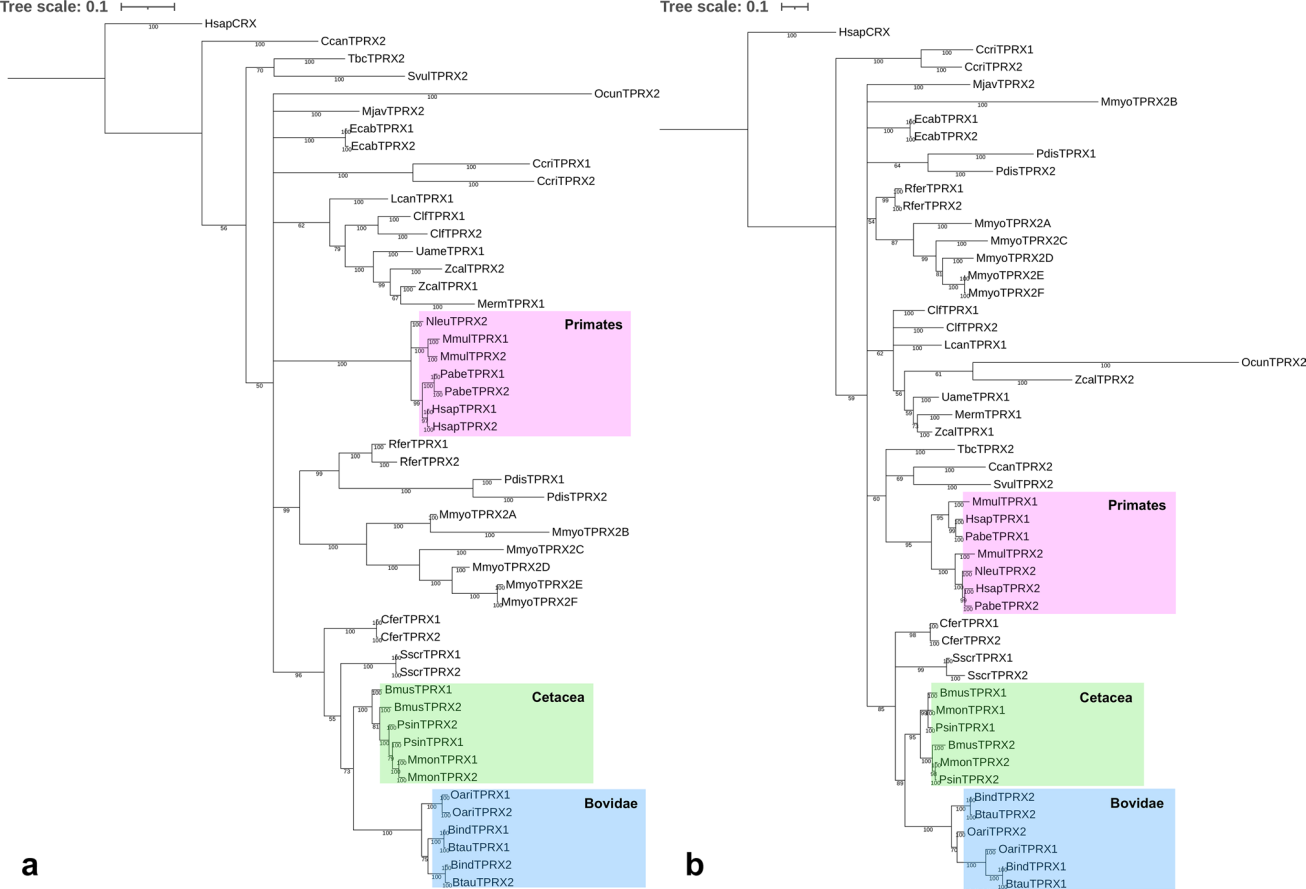
Bayesian phylogenies inferred using partition 1 (**a**) and partition 2 (**b**) of putatively functional *TPRX1* and *TPRX2* genes split at the gene conversion breakpoint identified by GARD. Boxes highlight the Bovidae, Cetacea and Primates, where topology differs markedly between the two trees. For example, Tree b is consistent with a gene conversion event at the base of the Primates; Tree a has conspecific pairs of *TPRX* genes consistent with additional more recent gene conversion events in the ancestors of these species within the Primates. Species abbreviations as in Fig. 2

Gene conversion occurring repeatedly in one region of a gene pair is predicted to result in differences between phylogenetic trees built from different sub-regions of the genes. Using GARD, which tests for phylogenetic incongruence within a gene (Kosakovsky Pond et al. 2006a, b), we identified a consistent putative gene conversion breakpoint, located immediately downstream of the homeobox, dividing the gene into two regions with different phylogenetic histories (null model AIC_C_ = 54,094.1, breakpoint model AIC_C_ = 52,467.8, ΔAIC_C_ = 1626.3; null model Akaike weight (w_i_) = 0, breakpoint model Akaike weight (w_i_) = 1; breakpoint model receives 100% of the weight of the models compared). Bayesian nucleotide sequence phylogenies of partition 1 (including the homeobox) and partition 2 (downstream) show different topologies (Fig. 6; quartet distance = 41,506; quartet divergence = 0.102). Partition 1 trees show more gene conversion events than partition 2 (e.g. Bovidae, Cetacea and Primates in Fig. 6), reinforcing the hypothesis that the 5’ region is more prone to gene conversion.

Finally, we used GENECONV (Sawyer 1989) to search for long regions of unusually high sequence identity in multiple sequence alignments as further evidence of gene conversion. In the 53 TPRX sequences analysed, GENECONV identifies eight gene conversion events by global comparisons (Online Resource Table S5) and 613 fragments by pairwise comparisons (Online Resource Table S6), ranging from 9 to 492 bp in length. Pairwise comparisons are particularly powerful for detecting very recent gene conversion. For example, we find evidence for ten ‘species-specific’ gene conversion events (Online Resource Table S7), eight of which were also detected by phylogenetic methods as forming pairs in the homeobox-only tree (Fig. 4b). Notably, GENECONV and GARD give similar locations for the position of gene conversion breakpoints between 5’ and 3’ regions, and both show that the upstream region is subject to more frequent gene conversion than the downstream region. Across all species, no fragments have GENECONV breakpoints downstream of position 492 of the 2331 bp multiple sequence alignment (Online Resource Fig. S5); in the majority of species, position 492 is very close to the putative gene conversion breakpoint identified by GARD, and in human they are only nine nucleotides apart (a large insertion in bats means that they are further apart in the full alignment, Online Resource Fig. S6). This corroboration by two methods lends strong support to this partition, which is within exon 3, downstream of the homeobox.

We note that gene conversion continued to occur between the six *My. myotis TPRX2* duplicates following tandem duplication, with 13 fragments identified by GENECONV (Online Resource Table S6). Furthermore, gene conversion in the ETCHbox genes is not limited to *TPRX*. Pairwise analysis using GENECONV identifies 15 gene conversion events between *Mi. murinus LEUTX* tandem duplicates and 367 events between *O. cuniculus LEUTX* duplicates (Online Resource Table S8). Both results are reinforced by GARD (Online Resource Table S9).

### Positive Selection in ETCHbox Genes

Using Tajima’s relative rate test (Tajima 1993), we find that all ETCHbox sequences have a faster evolutionary rate than their sister gene *CRX* (124 genes analysed; Online Resource Table S10). To investigate if the elevated evolutionary rates are due to positive selection, we used BUSTED (Murrell et al. 2015) to test for episodes of selection that may vary over time and between lineages, and MEME (Murrell et al. 2012) to identify specific sites under selection. These analyses were performed on complete coding sequences of *ARGFX*, *DPRX*, *LEUTX*, *PARGFX* and *TPRX*. However, since gene conversion leads to phylogenetic incongruity, which interferes with detecting positive selection (Anisimova et al. 2003; Shriner et al. 2003; Kosakovsky Pond et al. 2006b), we divided *TPRX* genes at the gene conversion breakpoint identified by GARD into 5’ and 3’ regions and performed analyses separately on the two regions.

**Fig. 7:**
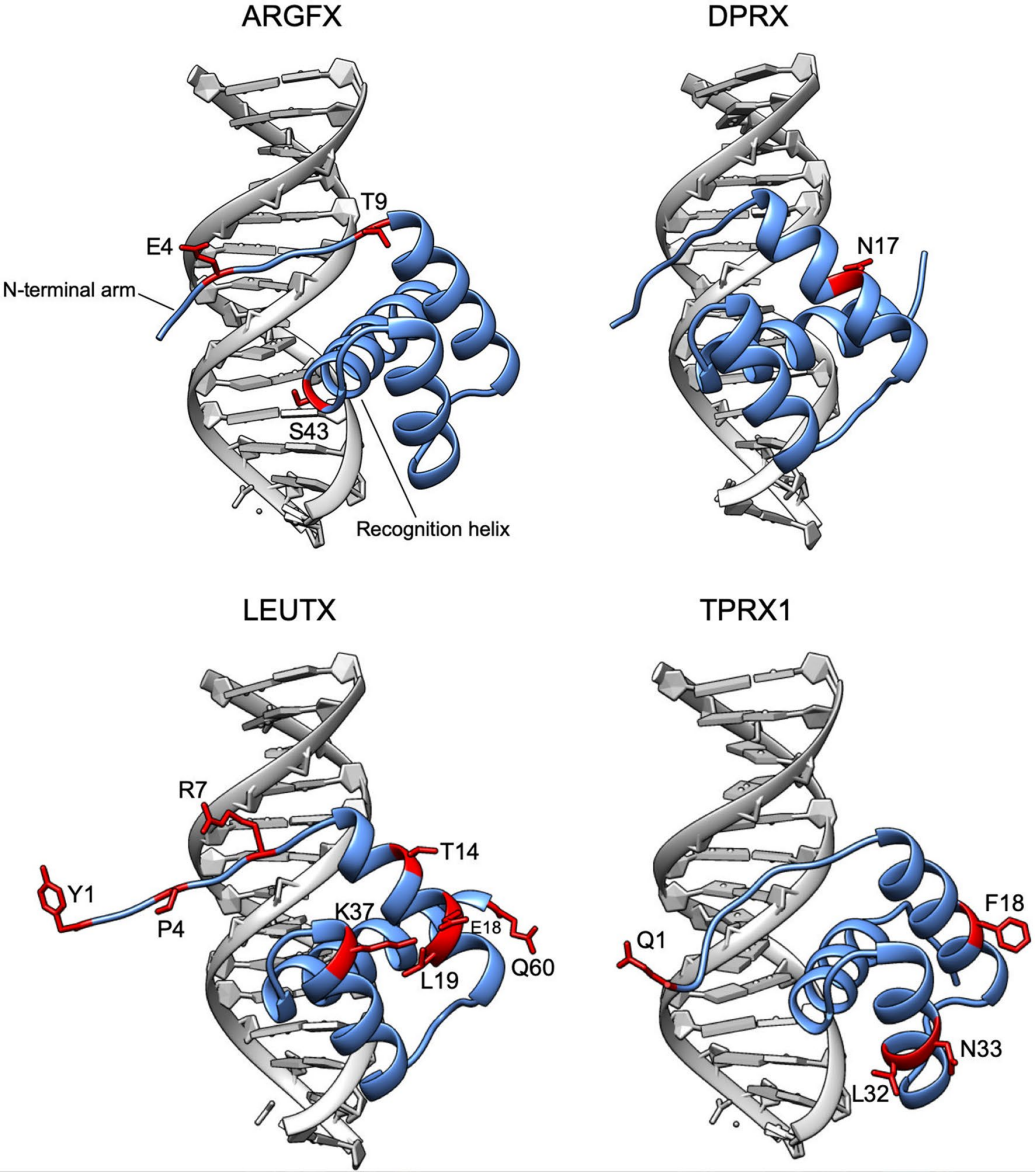
Models of ETCHbox homeodomain structures including sites under positive selection. Homeodomains of human ETCHbox proteins (blue) are modelled in complex with DNA (grey). Residues under positive selection are coloured red. Amino acid side chains are shown for sites under positive selection only. Letters show the identity of positively selected residues in human, numbers show their position within the homeodomain. TPRX1 and TPRX2 homeodomains are identical due to gene conversion so only one is shown

Using BUSTED (Murrell et al. 2015), we detect evidence of episodic positive selection during the evolution of *ARGFX*, *DPRX*, *LEUTX*, *PARGFX* and both partitions of the *TPRX* genes (LRT *p* < 0.05 for all genes). We also find strong evidence for positive selection in *ARGFX*, *DPRX*, *LEUTX*, *TPRX* partition 1 and *TPRX* partition 2, but not *PARGFX*, using CODEML (Yang 1997, 2007) (Online Resource Table S11), supporting the BUSTED result. Using MEME (Murrell et al. 2012), we find evidence for positive selection acting on between 3 (*PARGFX*) and 31 (*TPRX*) codons in each gene (Online Resource Table S12). The sites deduced to have undergone positive selection are spread across the encoded proteins, and include codons within homeodomains (*ARGFX* 3 sites; *DPRX* 1 site; *LEUTX* 8 sites; *TPRX* 4 sites; Online Resource Fig. S7). The spatial locations of sites under positive selection within homeodomains were inferred by comparative modelling of human ETCHbox homeodomains to a known PRD-class structure using Modeller (Šali and Blundell 1993) (Fig. 7, Online Resource Fig. S8). Sites under positive selection include those within the N-terminal arm of ARGFX (E4), LEUTX (Y1, P4, R7) and TPRX1/2 (Q1), and the recognition helix of ARGFX (S43).

Using RELAX (Wertheim et al. 2015), we also find evidence for relaxed selection in all ETCHbox genes compared with their sister gene *CRX* (Online Resource Table S13), suggesting that a combination of relaxed and positive selection is required to explain the fast evolutionary rate of these genes.

## Discussion

After duplication from *CRX* in the lineage leading to eutherians, the Eutherian Totipotent Cell Homeobox (ETCHbox) genes underwent asymmetric evolution and continued to be duplicated and lost (Maeso et al. 2016). The genes are suspected to have important roles in preimplantation development and embryonic genome activation (Jouhilahti et al. 2016; Maeso et al. 2016), making observed variability of ETCHbox gene sets a mystery. Here, we compared the ETCHbox complements of 34 species with the aim of illuminating the processes that have sculpted such varied repertoires. Restricting the analyses to genomes sequenced using long-read technologies allowed us to establish with confidence clear examples of gene duplication and secondary loss, something that was challenging in previous work based on lower quality genome assemblies.

We find that, despite extensive and frequent gene loss, all sampled species possess at least two putatively functional ETCHbox genes. This retention suggests that the genes, collectively, are indispensable for eutherian development, and that fluctuations in gene number and rapid sequence evolution are not due to the lack of a function and neutrality. Previous work has shown that some ETCHbox genes can act in an antagonistic fashion, with gene sets upregulated by one gene overlapping with those downregulated by another (Jouhilahti et al. 2016; Maeso et al. 2016). This antagonism could explain why at least two different genes are always retained.

A second line of evidence supporting functionality is that all of the genes have been under recent positive selection, including at residues within the homeodomain. Residues in the N-terminal arm of the ARGFX, LEUTX and TPRX1 homeodomains, identified as having amino acid change driven by positive selection, are suggested by comparative modelling to interact with the minor groove of DNA. Residue 7 specifically, deduced to be under selection in LEUTX, is involved in sequence-specific contact in other homeodomain proteins and therefore may affect binding specificity (Ekker et al. 1994; Damante et al. 1996). ARGFX homeodomain residue S43, also deduced to have been under positive selection, sits within the DNA-binding and specificity-determining recognition helix of PRD-class homeodomains (Bruun et al. 2005). These results suggest that there has been selection for altered DNA-binding properties in ETCHbox homeodomains. In addition, residues in homeodomain helices 1 and 2 are deduced to have been under selection in DPRX, LEUTX and TPRX proteins; modelling suggests that most of these residues are on the outer surface of the homeodomain. Since both of these helices have been proposed to mediate protein–protein interactions in some homeodomains, including those of the PRD class (Wilson et al. 1995; Simon et al. 1997; Zaffran and Frasch 2005; Plaza et al. 2008; Altamirano-Torres et al. 2018), we propose that this selection has altered ETCHbox protein–protein binding properties. Madissoon et al. (2016) found that homeodomain differences are not sufficient to explain the differing transcriptional effects of PRD-like genes, implying that other protein domains also contribute to specificity; we therefore suggest that sites outside of the homeodomain that are under selection also influence specificity. Overall, our results suggest that there has been on-going and divergent selection for altered DNA-binding specificity and/or co-factor interactions in the ETCHbox genes, implying that functions have been modified as part of their rapid evolution during mammalian radiation. Experimental evidence supports these conclusions. For example, Royall et al. (2018) found that, at some point during rodent evolution, *Crxos* (*TPRX1*) likely underwent a change in function to take on part of the role of *ARGFX.*

Though positive selection has contributed to changes in ETCHbox protein sequences, their timing of expression during development has remained relatively stable. Consistent with the results of Maeso et al. (2016), we find that all sampled species possess at least one ‘processed pseudogene’ derived from an ETCHbox gene; these are generated by retrotransposition exclusively from genes expressed in the germline, including uncommitted early embryonic cells (Vanin 1985; Maestre et al. 1995). This suggests that across large phylogenetic distances the ETCHbox genes retain expression in the very early embryo. This is corroborated by transcriptome data which showed that ETCHbox genes are expressed in preimplantation development in both humans (Euarchontoglires) and cattle (Laurasiatheria) (Maeso et al. 2016).

Despite all eutherian mammals possessing at least two ETCHbox genes, there has been extensive gene loss. We find that of the six ETCHbox genes (*ARGFX*, *DPRX*, *LEUTX*, *PARGFX*, *TPRX1*, *TPRX2*), each has been lost in at least one sampled species, with *PARGFX* lost at the highest rate; furthermore, all sampled species have lost at least one ETCHbox gene. This pattern could be explained through a degree of genetic functional redundancy, whereby multiple genes perform similar functions and can partially substitute for each other, a pattern common after gene duplication (Wagner 1996; Kafri et al. 2009; Zhang 2012). Functional overlap could lead to relaxed selection, allowing repertoires to vary while an overall indispensable function is maintained. This suggestion is consistent with the finding of Maeso et al. (2016) that gene sets regulated by *LEUTX* and *TPRX1* in human cells have a large degree of overlap. Partial redundancy between ETCHbox genes would not be without precedent: it is a common component of biological systems and is known to be a feature of other homeobox duplicates, including members of different *HOX* clusters in mammals (McNulty et al. 2005; Tvrdik and Capecchi 2006; Kafri et al. 2009; Ruff et al. 2015). Genetic redundancy can be evolutionarily stable and may be maintained by selection when, for example, one of the genes occasionally fails to perform a function successfully, or when genes possess other, non-redundant functions which are co-selected with redundant ones (Nowak et al. 1997; Vavouri et al. 2008; Kafri et al. 2009).

Gene duplication is a potential driver of functional innovation. Here we identify large tandem arrays of ETCHbox duplicates in several species, including *O. cuniculus* and *Mi. murinus LEUTX* and *P. leucopus* and *My. myotis TPRX2.* Further arrays have been previously described, such as the 66 Obox (*TPRX2*) loci of *Mu. musculus* (Maeso et al. 2016; Royall et al. 2018). It is likely that the propensity for tandem duplication stems from the position of these genes in a dynamic and unstable genomic region (chromosome 19 in human, 18 in *B. taurus*), in which there is a high density of repetitive sequences, low density of recombination hotspots and elevated gene duplication rates (Castresana 2002; Grimwood et al. 2004; Myers et al. 2005; Maeso et al. 2016), but the selective forces favouring retention of these duplicates are currently unclear. There are three main mechanisms by which duplications could be advantageous in the short term (Innan and Kondrashov 2010): (1) by increasing gene dosage where function is dosage sensitive (Kondrashov and Koonin 2004); (2) by buffering against deleterious mutations (Haldane 1933; Gu et al. 2003); and (3) the immediate emergence of a new function, for example due to the partial duplication of regulatory elements, or alteration of genomic location (Lercher et al. 2003; Lynch and Katju 2004; Katju and Lynch 2006). None of these explanations appears sufficient to explain the giant arrays observed for ETCHbox genes. The alternative is that the initial duplication event is selectively neutral (Innan and Kondrashov 2010), but duplicates are retained following either neofunctionalisation (Ohno 1970) or duplication–degeneration–complementation (DDC) (Force et al. 1999). Current data support this model. The high rates of pseudogenisation in the tandem arrays (45% for *O. cuniculus LEUTX*, 79% for *P. leucopus TPRX2*) suggest that some duplicates are selectively neutral and not actively retained, and previous studies have uncovered functional differences between *Mu. musculus Obox* duplicates, implying that sub- or neofunctionalisation has occurred following expansion of the tandem array (Royall et al. 2018).

Tandem gene duplicates can be subject to gene conversion, and we find overwhelming support that gene conversion has been a major force affecting *TPRX1* and *TPRX2* molecular evolution throughout the Boreoeutheria. Interestingly, these two genes are not directly adjacent to each other, but lie either side of the *CRX* locus. Gene conversion is expected to cause concerted evolution, meaning that instead of gene duplicates accumulating mutations independently they evolve in parallel, maintaining a higher than expected level of sequence similarity (Ohta 1980; Zimmer et al. 1980; Arnheim 1983; Sugino and Innan 2005; Fawcett and Innan 2011). Gene conversion thus restricts the ability of duplicates to neofunctionalise, because their sequence is repeatedly homogenised and divergence is lost (Innan 2003; Teshima and Innan 2008; Fawcett and Innan 2011; Korunes and Noor 2017). As genes diverge, the accumulation of many small mutations or fewer large sequence changes (e.g. transposable element insertion) can cause a threshold to be reached, at which point sequences differ enough that gene conversion no longer occurs; at this stage, independent evolution commences and neofunctionalisation may take place (Walsh 1987; Teshima and Innan 2008; Fawcett and Innan 2011). The recent gene conversion events and high sequence similarities detected in this work suggest that this threshold is yet to be reached in the *TPRX* genes of most sampled lineages.

It is interesting to consider why *TPRX1* and *TPRX2* seem subject to such frequent gene conversion events, and why this has continued over long time periods across diverse lineages. One possibility is that the genes are dosage-sensitive with a beneficial effect if dosage is increased, as this can cause gene conversion to be favoured by selection (Sugino and Innan 2006). We suggest that gene conversion will affect the strength of selection on *TPRX* genes, whether it be directional or balancing (Fawcett and Innan 2011). For example, gene conversion can lead to faster adaptation because alleles can be transferred between paralogues, enabling the spread of beneficial mutations and elimination of deleterious ones (Winderickx et al. 1993; Chen et al. 2007; Mano and Innan 2008; Korunes and Noor 2017). It is also expected to lead to faster adaptation through increasing effective population size, which enhances the efficiency of selection occurring within a gene family (Mano and Innan 2008). Overall, gene conversion has been a critical factor driving *TPRX1* and *TPRX2* evolution and is predicted to have a dramatic influence on their functional role.

## Conclusion

The ETCHbox genes represent an example of the recruitment of eutherian mammal-specific homeobox genes to a very early developmental stage, making them a promising model to study the evolution of young, lineage-specific homeobox genes. Our data show that, unlike the vast majority of homeobox genes, they have been subject to frequent tandem duplications and gene losses over relatively short evolutionary timescales, leading to varied ETCHbox repertoires even amongst closely related species. This includes newly discovered large tandem arrays of homeobox genes. The data also suggest that the ETCHbox genes are indispensable to eutherian preimplantation development, and that positive selection has continued to modify their functions. Finally, we show that gene conversion between *TPRX1* and *TPRX2* has occurred on a striking number of occasions and prevented divergence of their homeodomains; the consequences of this for function are currently unclear. Overall, high rates of gene duplication and loss, extensive divergence, concerted evolution and positive selection have sculpted the varied ETCHbox repertoires that are observed across eutherians; our results support the idea that antagonism and redundancy are key factors in determining these unusual evolutionary patterns.

## Supplementary Information

Below is the link to the electronic supplementary material.

Supplementary file1 (XLSX 95 KB)

Supplementary file2 (PDF 33801 KB)

## Data Availability

The datasets supporting the conclusions of this article are included within the article and its Electronic Supplementary Material.
