# Supplementary material for: Dynamic Molecular Evolution of Mammalian Homeobox Genes: Duplication, Loss, Divergence and Gene Conversion Sculpt PRD Class Repertoires
Source: J Mol Evol. 2021 Jun 7;89(6):396–414. doi: 10.1007/s00239-021-10012-6 (PMC8208926; doi:10.1007/s00239-021-10012-6)
Supplement: Supplementary file 2 — Supplementary file2 (PDF 33801 KB) [file 239_2021_10012_MOESM2_ESM.pdf]

Journal of Molecular Evolution

Dynamic molecular evolution of mammalian homeobox genes:
duplication, loss, divergence and gene conversion sculpt PRD class repertoires

Thomas D. Lewin[1], Amy H. Royall[1] and Peter W. H. Holland[1*]

Affiliations:

[1]Department of Zoology, University of Oxford, 11a Mansfield Road, Oxford, OX1 3SZ, UK

[*]Corresponding author: Peter W. H. Holland (peter.holland@zoo.ox.ac.uk)

Electronic Supplementary Material 3: Online Resource Fig. S1 – S8

Online Resource – Fig. S1

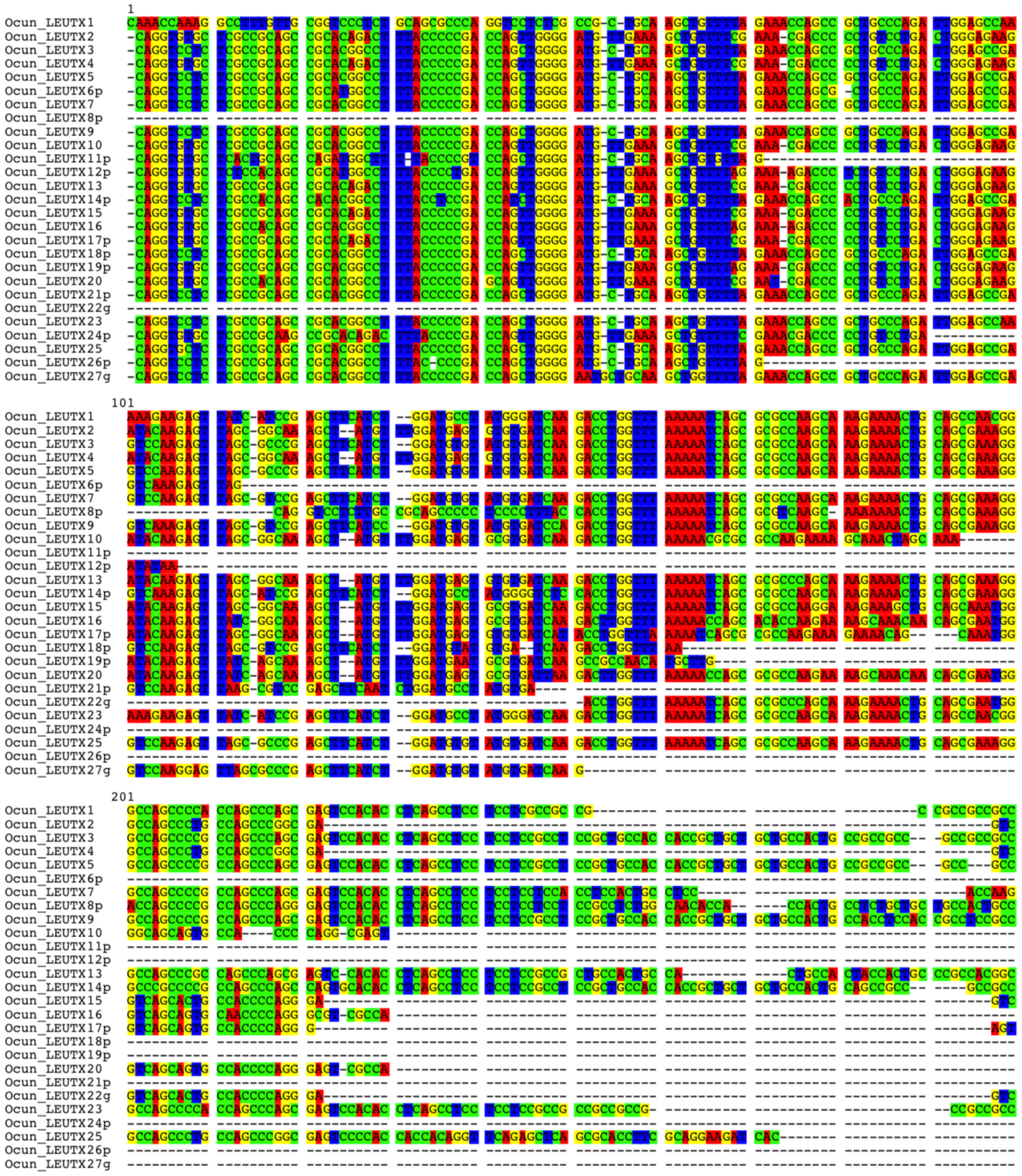

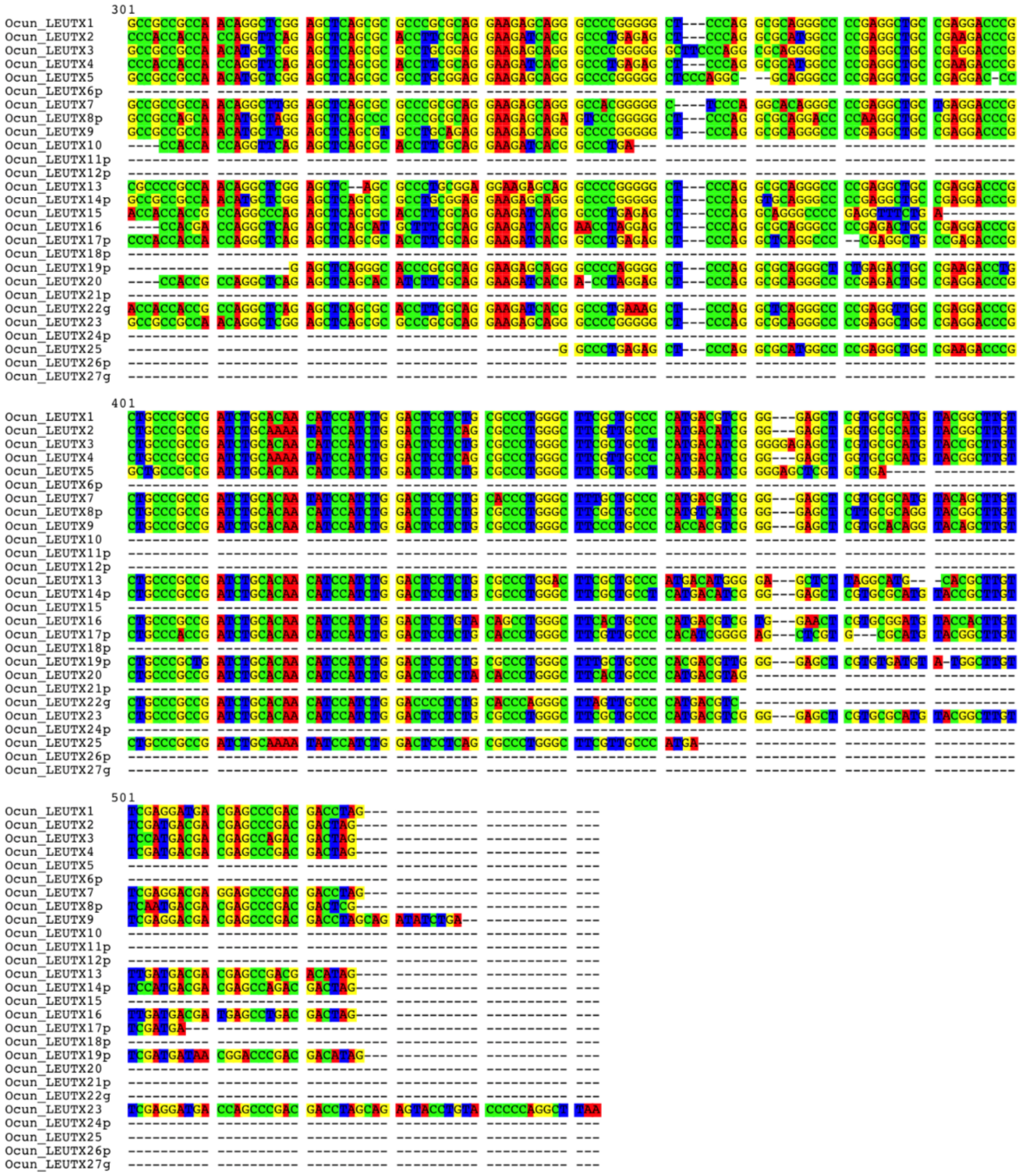

Online Resource – Fig. S1 Multiple sequence alignment of *Oryctolagus cuniculus LEUTX* genes. Homeobox-containing exons (coding exons 2 and 3) shown; coding sequence shown only. The following genes are designated pseudogenes (no complete homeobox): *LEUTX6p* (frameshift), *LEUTX8p* (missing part of exon 2), *LEUTX11p* (frameshift; splice site lost), *LEUTX12p* (stop codon in homeobox), *LEUTX14p* (splice site lost), *LEUTX17p* (frameshift), *LEUTX18p* (frameshift), *LEUTX19p* (missing part of exon 3), *LEUTX21p* (frameshift), *LEUTX24p* (frameshift), *LEUTX26p* (frameshift). The status of *LEUTX22g* and *LEUTX27g* is unknown due to assembly gaps. p = pseudogene, g = assembly gaps at locus.

Online Resource – Fig. S2

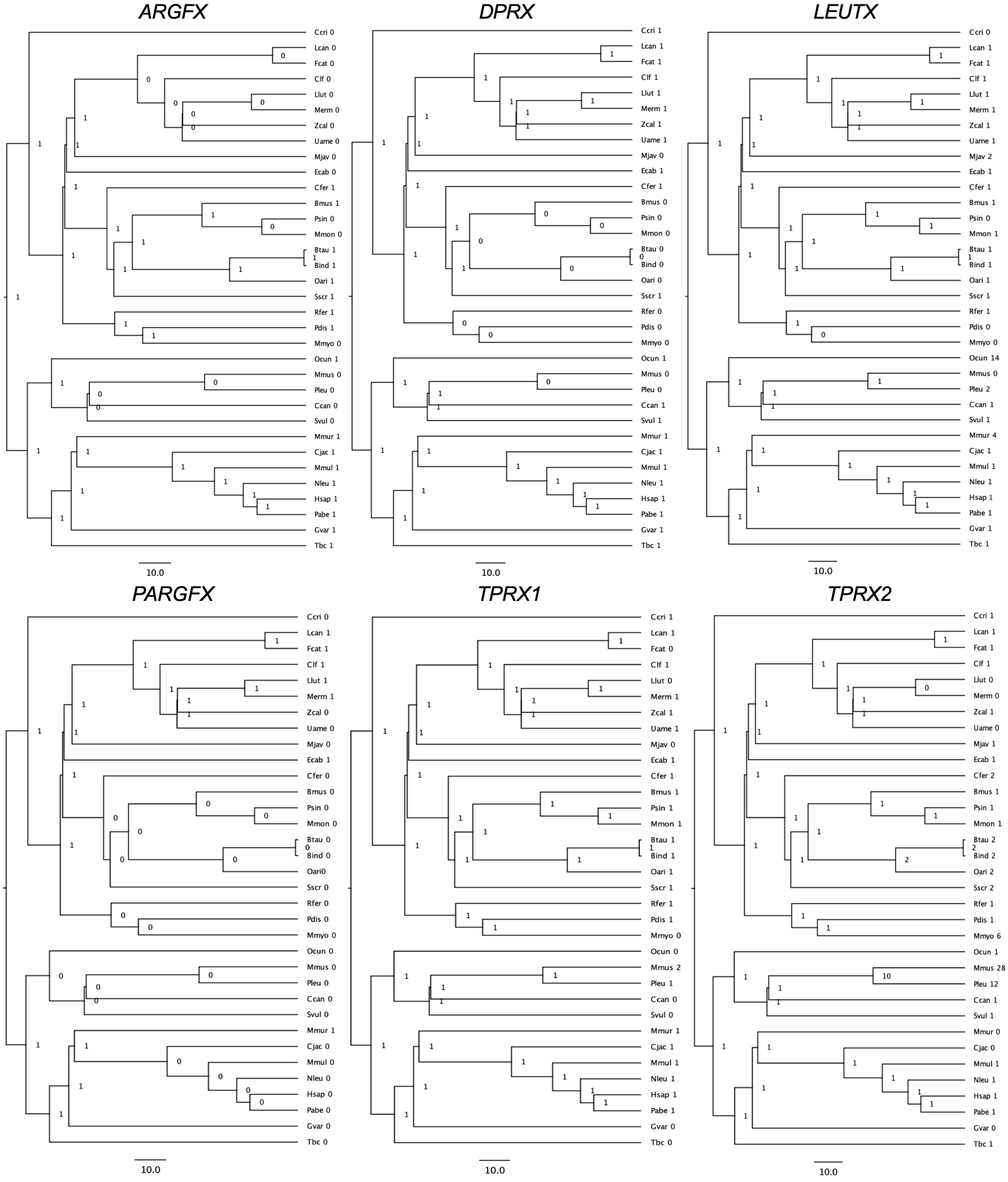

Online Resource – Fig. S2 Phylogenies of 34 eutherian species with values indicating the number of gene copies present. Gene copy numbers at internal nodes were estimated using CAFE (De Bie et al. 2006; Han et al. 2013). Putative pseudogenes are excluded. The phylogeny for this analysis was calculated using TimeTree (Kumar et al. 2017). Species abbreviations as in Fig. 2 of main text.

Online Resource – Fig. S3

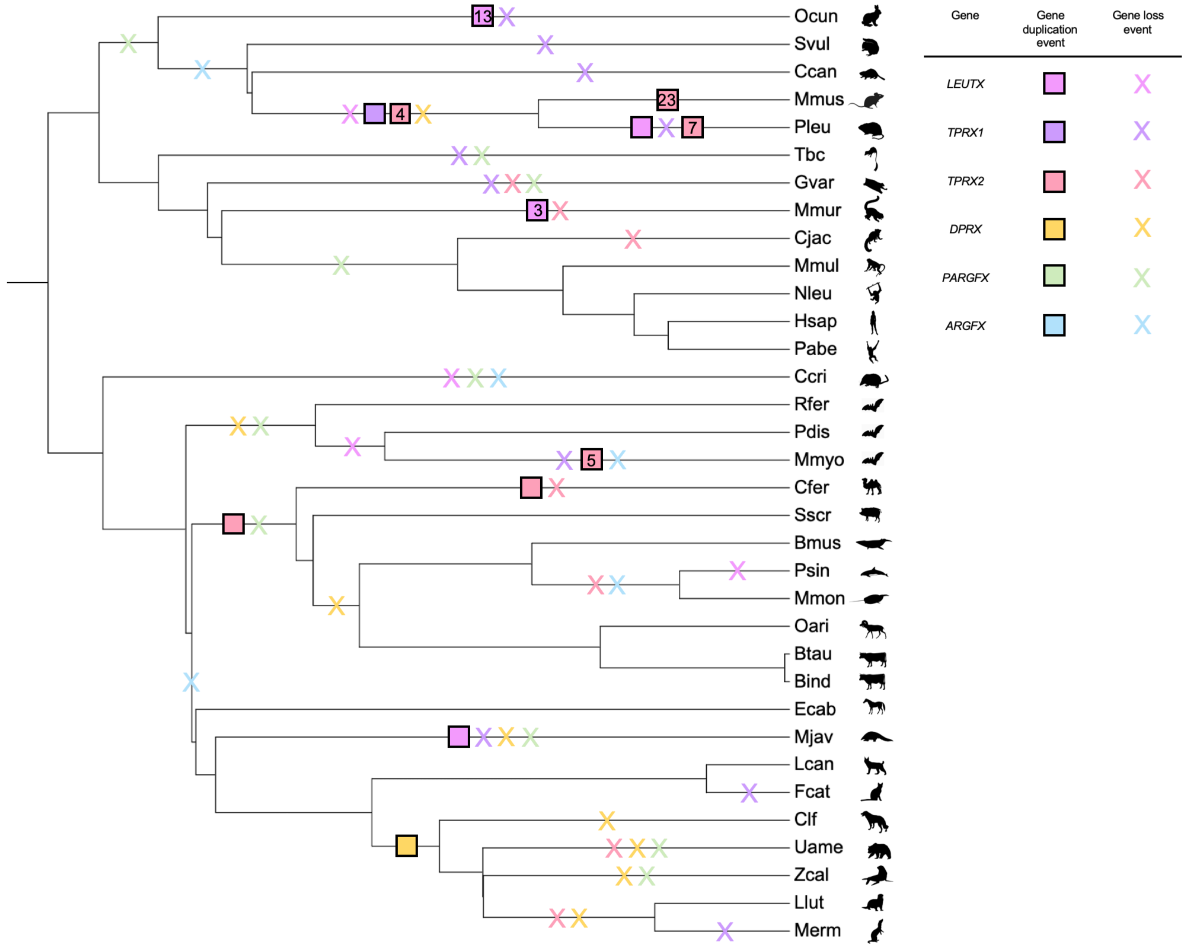

Online Resource – Fig. S3 Phylogeny of 34 eutherian species including gene duplication and gene loss events. Lineages where a gene duplication has occurred are marked with a coloured square, lineages where a gene loss event has occurred are marked with a coloured X. Pink = *LEUTX*, purple = *TPRX1*, red = *TPRX2*, yellow = *DPRX*, green = *PARGFX*, blue = *ARGFX*. Gene duplication and loss events were estimated using Notung (Chen et al. 2000; Durand et al. 2006). Putative pseudogenes are excluded. The phylogeny for this analysis was calculated using TimeTree (Kumar et al. 2017). Species abbreviations as in Fig. 2 of main text.

Online Resource – Fig. S4

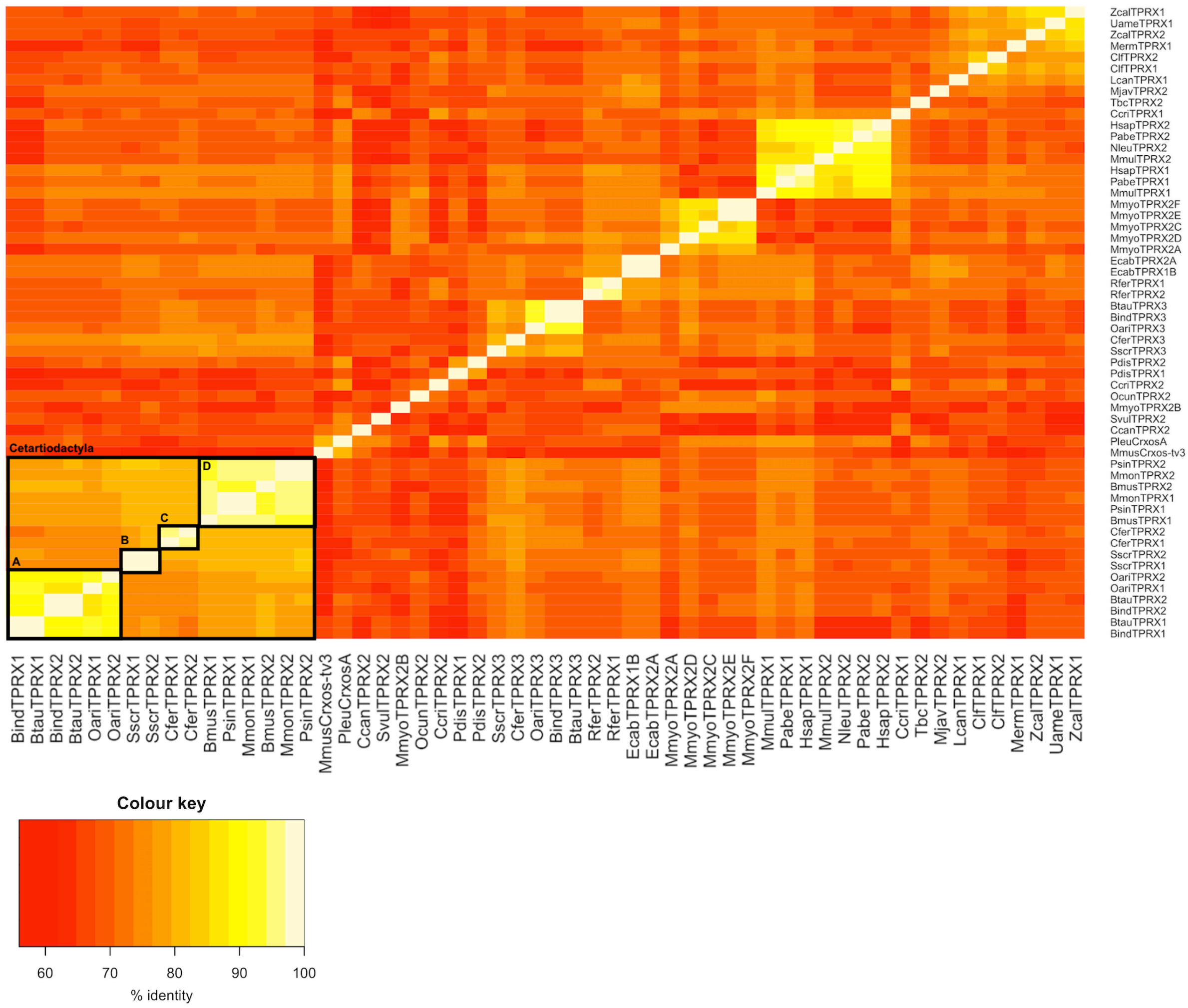

Online Resource – Fig. S4 Heatmap of percent nucleotide sequence identity between pairwise alignments of *TPRX1*, *TPRX2* and *TPRX3* (even-toed ungulate-specific) coding regions, with sequences clustered by similarity. Lighter colours indicate higher levels of identity; red indicates lowest identity. High similarity between *TPRX1* and *TPRX2* genes of one species is indicative of recent gene conversion events (e.g *Rhinolophus ferrumequinum*, Rfer and *Equus caballus*, Ecab). Box 'Cetartiodactyla' highlights the Cetartiodactyla genes. All Cetartiodactyla *TPRX1* and *TPRX2* genes are more similar to each other than to any other species' genes (indicated by brighter colours in the heatmap), suggesting a gene conversion event at the base of the Cetartiodactyla. Inside this Cetartiodactyla box, there are further bright boxes representing the *TPRX1* and *TPRX2* genes of the Bovidae (box A), *Sus scrofa* (Sscr; B), *Camelus ferus* (Cfer; C) and Cetacea (D), suggesting that further gene conversion events have occurred in these lineages within the Cetartiodactyla. Species abbreviations as in Fig. 2 of main text.

Online Resource – Fig. S5

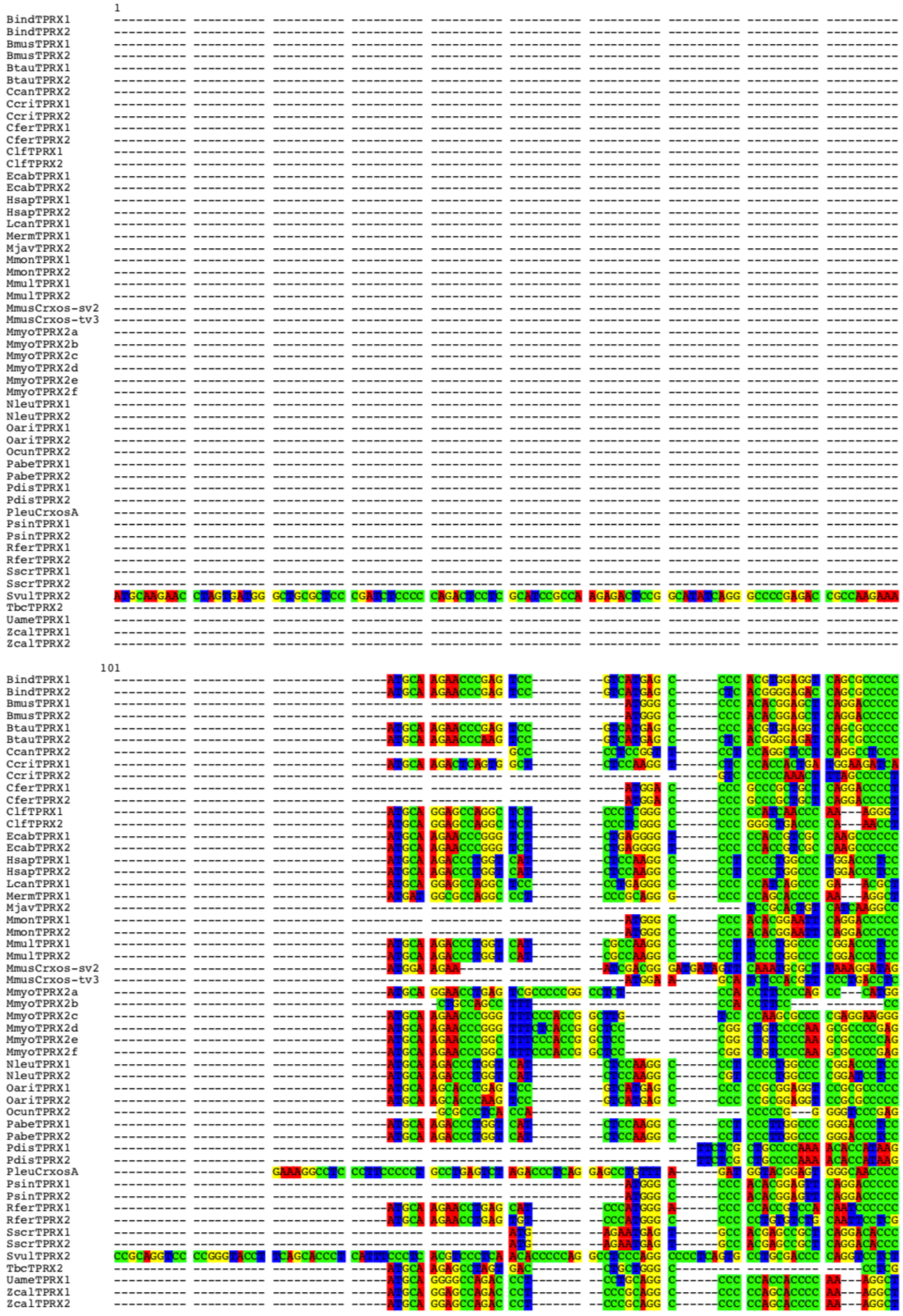

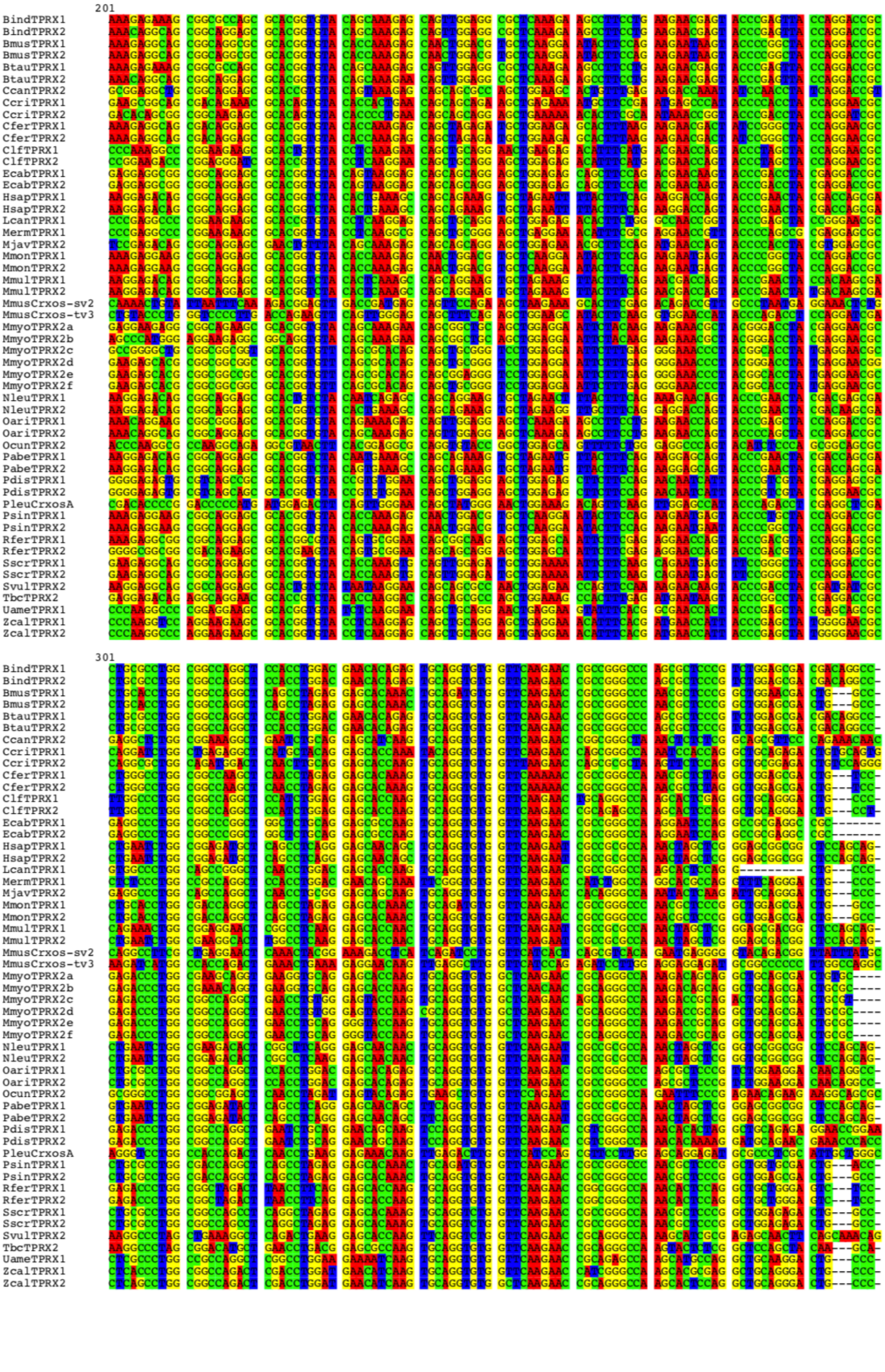
201
BindTPRX1
BindTPRX2
BmusTPRX1
BmusTPRX2
BtauTPRX1
BtauTPRX2
CcanTPRX2
CcriTPRX1
CcriTPRX2
CferTPRX1
CferTPRX2
ClfTPRX1
ClfTPRX2
EcabTPRX1
EcabTPRX2
HsapTPRX1
HsapTPRX2
LcanTPRX1
MermTPRX1
MjavTPRX2
MmonTPRX1
MmonTPRX2
MmulTPRX1
MmulTPRX2
MmusCrxos-sv2
MmusCrxos-tv3
MmyoTPRX2a
MmyoTPRX2b
MmyoTPRX2c
MmyoTPRX2d
MmyoTPRX2e
MmyoTPRX2f
NleuTPRX1
NleuTPRX2
OariTPRX1
OariTPRX2
OcunTPRX2
PabeTPRX1
PabeTPRX2
PdisTPRX1
PdisTPRX2
PleuCrxosA
PsinTPRX1
PsinTPRX2
RferTPRX1
RferTPRX2
SscrTPRX1
SscrTPRX2
SvulTPRX2
TbcTPRX2
UameTPRX1
ZcalTPRX1
ZcalTPRX2
301
BindTPRX1
BindTPRX2
BmusTPRX1
BmusTPRX2
BtauTPRX1
BtauTPRX2
CcanTPRX2
CcriTPRX1
CcriTPRX2
CferTPRX1
CferTPRX2
ClfTPRX1
ClfTPRX2
EcabTPRX1
EcabTPRX2
HsapTPRX1
HsapTPRX2
LcanTPRX1
MermTPRX1
MjavTPRX2
MmonTPRX1
MmonTPRX2
MmulTPRX1
MmulTPRX2
MmusCrxos-sv2
MmusCrxos-tv3
MmyoTPRX2a
MmyoTPRX2b
MmyoTPRX2c
MmyoTPRX2d
MmyoTPRX2e
MmyoTPRX2f
NleuTPRX1
NleuTPRX2
OariTPRX1
OariTPRX2
OcunTPRX2
PabeTPRX1
PabeTPRX2
PdisTPRX1
PdisTPRX2
PleuCrxosA
PsinTPRX1
PsinTPRX2
RferTPRX1
RferTPRX2
SscrTPRX1
SscrTPRX2
SvulTPRX2
TbcTPRX2
UameTPRX1
ZcalTPRX1
ZcalTPRX2

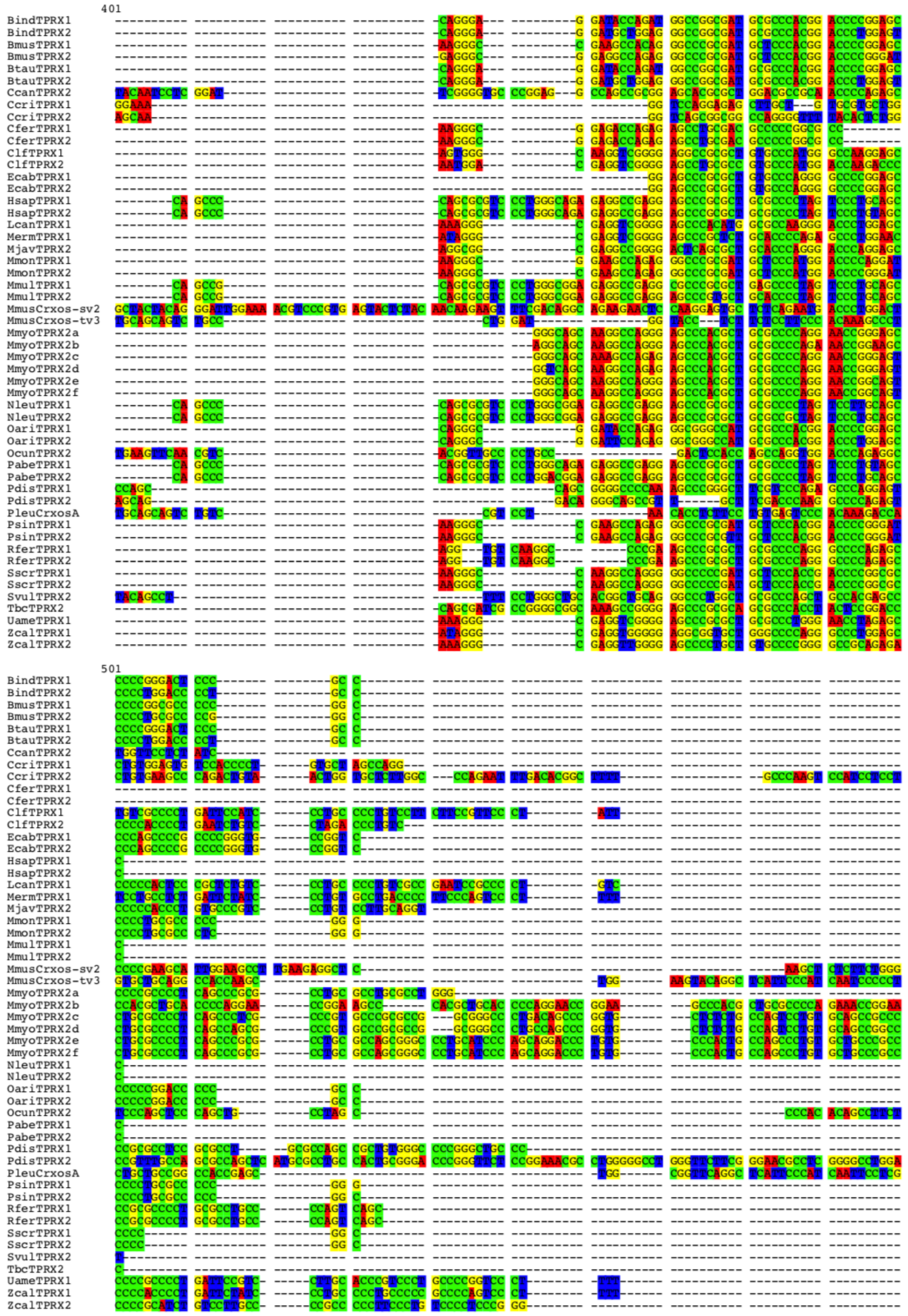
401
BindTPRX1
BindTPRX2
BmusTPRX1
BmusTPRX2
BtauTPRX1
BtauTPRX2
CcanTPRX2
CcriTPRX1
CcriTPRX2
CferTPRX1
CferTPRX2
ClfTPRX1
ClfTPRX2
EcabTPRX1
EcabTPRX2
HsapTPRX1
HsapTPRX2
LcanTPRX1
MermTPRX1
MjavTPRX2
MmonTPRX1
MmonTPRX2
MmulTPRX1
MmulTPRX2
MmusCrxos-sv2
MmusCrxos-tv3
MmyoTPRX2a
MmyoTPRX2b
MmyoTPRX2c
MmyoTPRX2d
MmyoTPRX2e
MmyoTPRX2f
NleuTPRX1
NleuTPRX2
OariTPRX1
OariTPRX2
OcunTPRX2
PabeTPRX1
PabeTPRX2
PdisTPRX1
PdisTPRX2
PleuCrxosA
PsinTPRX1
PsinTPRX2
RferTPRX1
RferTPRX2
SscrTPRX1
SscrTPRX2
SvulTPRX2
TbcTPRX2
UameTPRX1
ZcalTPRX1
ZcalTPRX2
501

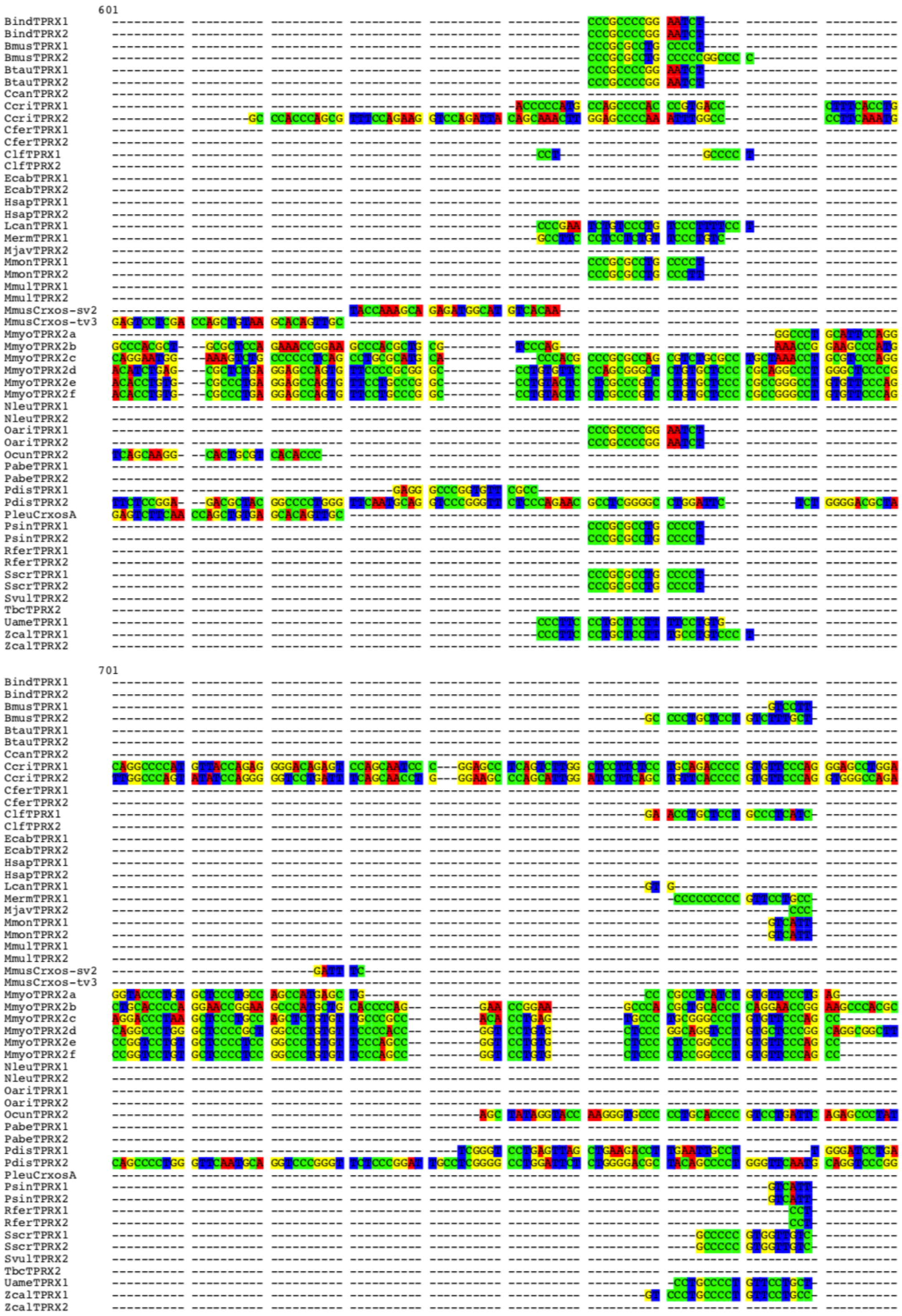
601
BindTPRX1
BindTPRX2
BmusTPRX1
BmusTPRX2
BtauTPRX1
BtauTPRX2
CcanTPRX2
CcriTPRX1
CcriTPRX2
CferTPRX1
CferTPRX2
ClfTPRX1
ClfTPRX2
EcabTPRX1
EcabTPRX2
HsapTPRX1
HsapTPRX2
LcanTPRX1
MermTPRX1
MjavTPRX2
MmonTPRX1
MmonTPRX2
MmulTPRX1
MmulTPRX2
MmusCrxos-sv2
MmusCrxos-tv3
MmyoTPRX2a
MmyoTPRX2b
MmyoTPRX2c
MmyoTPRX2d
MmyoTPRX2e
MmyoTPRX2f
NleuTPRX1
NleuTPRX2
OariTPRX1
OariTPRX2
OcunTPRX2
PabeTPRX1
PabeTPRX2
PdisTPRX1
PdisTPRX2
PleuCrxosA
PsinTPRX1
PsinTPRX2
RferTPRX1
RferTPRX2
SscrTPRX1
SscrTPRX2
SvulTPRX2
TbcTPRX2
UameTPRX1
ZcalTPRX1
ZcalTPRX2
701
BindTPRX1
BindTPRX2
BmusTPRX1
BmusTPRX2
BtauTPRX1
BtauTPRX2
CcanTPRX2
CcriTPRX1
CcriTPRX2
CferTPRX1
CferTPRX2
ClfTPRX1
ClfTPRX2
EcabTPRX1
EcabTPRX2
HsapTPRX1
HsapTPRX2
LcanTPRX1
MermTPRX1
MjavTPRX2
MmonTPRX1
MmonTPRX2
MmulTPRX1
MmulTPRX2
MmusCrxos-sv2
MmusCrxos-tv3
MmyoTPRX2a
MmyoTPRX2b
MmyoTPRX2c
MmyoTPRX2d
MmyoTPRX2e
MmyoTPRX2f
NleuTPRX1
NleuTPRX2
OariTPRX1
OariTPRX2
OcunTPRX2
PabeTPRX1
PabeTPRX2
PdisTPRX1
PdisTPRX2
PleuCrxosA
PsinTPRX1
PsinTPRX2
RferTPRX1
RferTPRX2
SscrTPRX1
SscrTPRX2
SvulTPRX2
TbcTPRX2
UameTPRX1
ZcalTPRX1
ZcalTPRX2

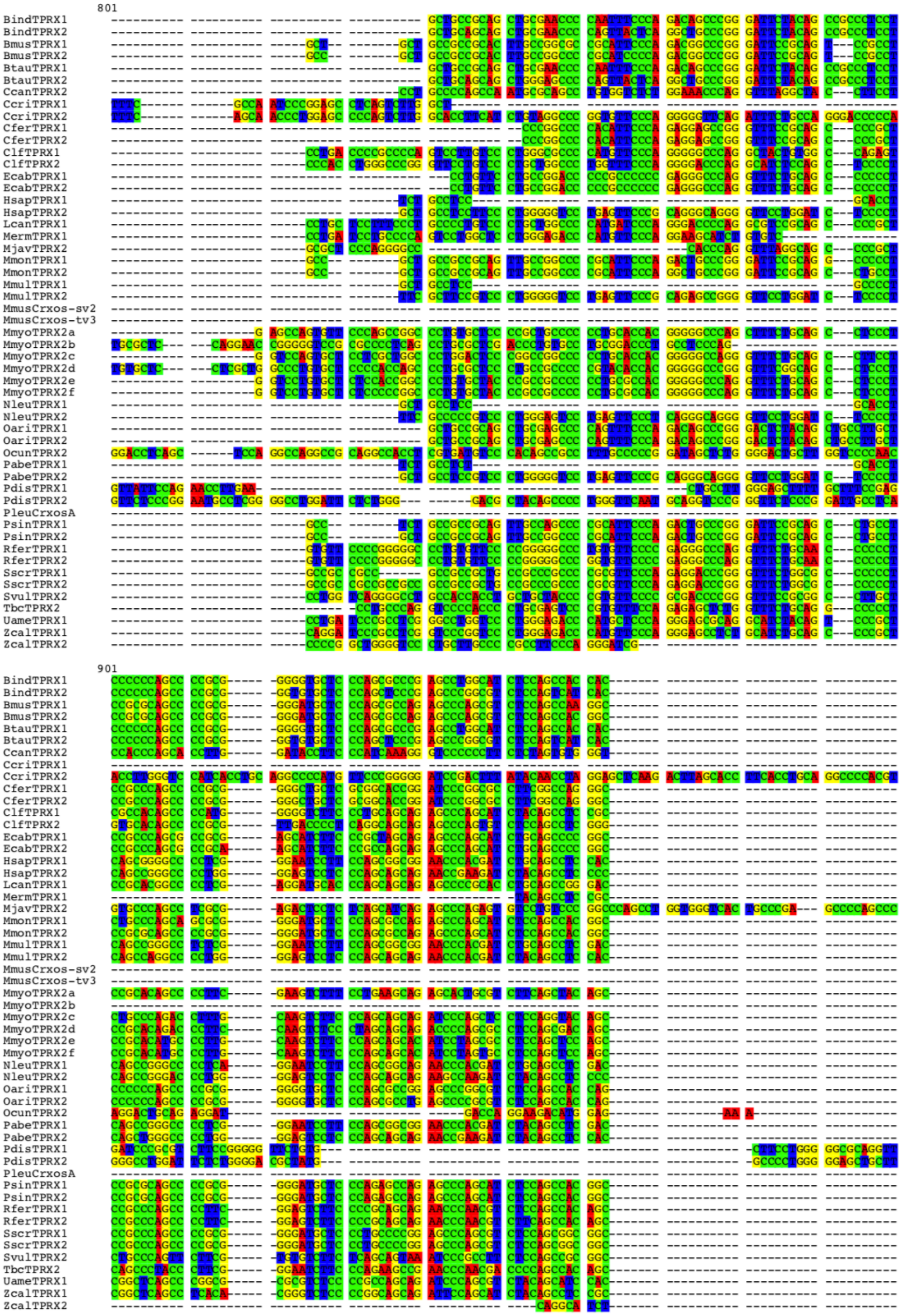

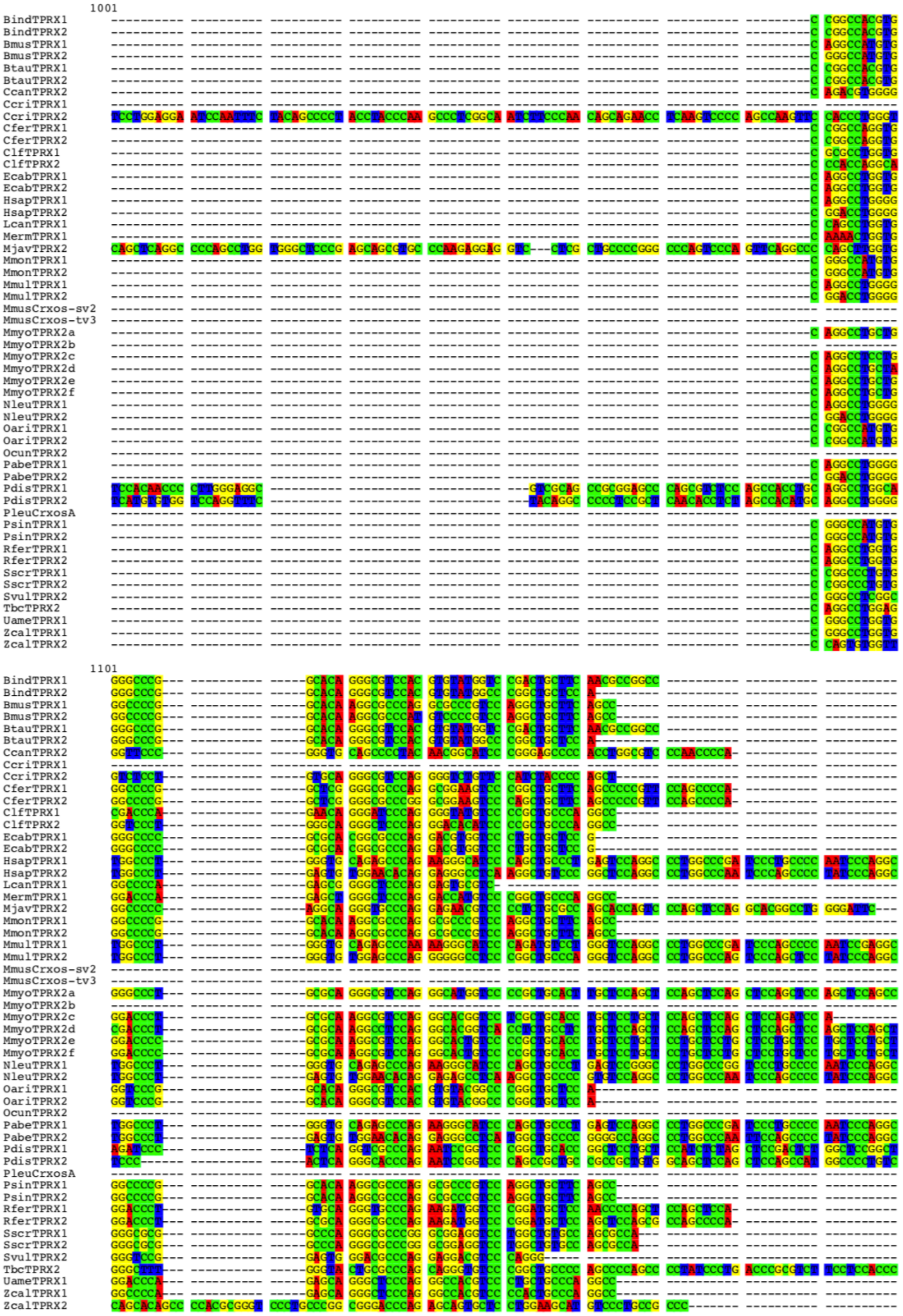

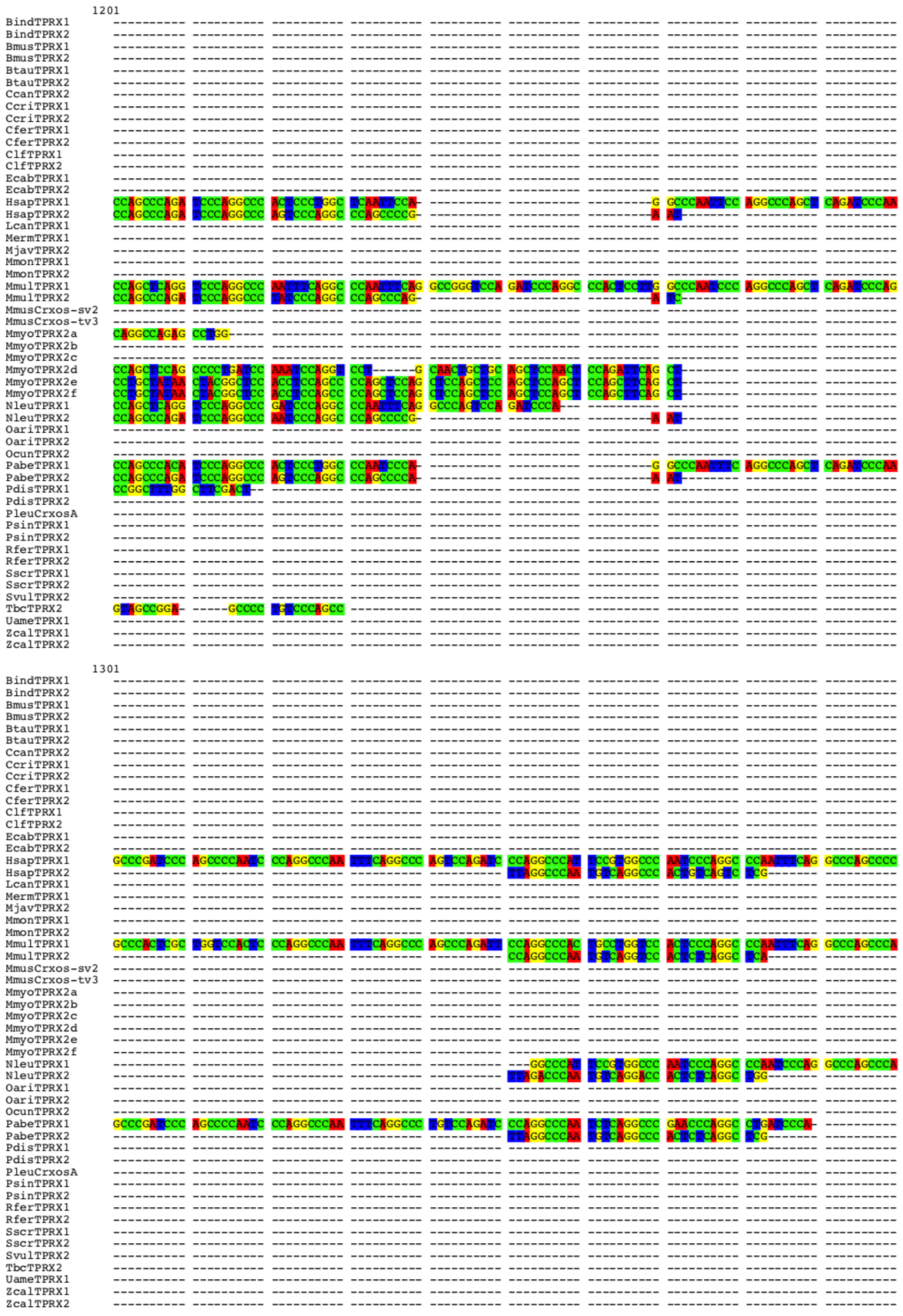

```
               1401
BindTPRX1      ---------- ---------- ---------- ---------- ---------- ---------- ---------- ---------- ---------- ----------
BindTPRX2      ---------- ---------- ---------- ---------- ---------- ---------- ---------- ---------- ---------- ----------
BmusTPRX1      ---------- ---------- ---------- ---------- ---------- ---------- ---------- ---------- ---------- ----------
BmusTPRX2      ---------- ---------- ---------- ---------- ---------- ---------- ---------- ---------- ---------- ----------
BtauTPRX1      ---------- ---------- ---------- ---------- ---------- ---------- ---------- ---------- ---------- ----------
BtauTPRX2      ---------- ---------- ---------- ---------- ---------- ---------- ---------- ---------- ---------- ----------
CcanTPRX2      ---------- ---------- ---------- ---------- ---------- ---------- ---------- ---------- ---------- ----------
CcriTPRX1      ---------- ---------- ---------- ---------- ---------- ---------- ---------- ---------- ---------- ----------
CcriTPRX2      ---------- ---------- ---------- ---------- ---------- ---------- ---------- ---------- ---------- ----------
CferTPRX1      ---------- ---------- ---------- ---------- ---------- ---------- ---------- ---------- ---------- ----------
CferTPRX2      ---------- ---------- ---------- ---------- ---------- ---------- ---------- ---------- ---------- ----------
ClfTPRX1       ---------- ---------- ---------- ---------- ---------- ---------- ---------- ---------- ---------- ----------
ClfTPRX2       ---------- ---------- ---------- ---------- ---------- ---------- ---------- ---------- ---------- ----------
EcabTPRX1      ---------- ---------- ---------- ---------- ---------- ---------- ---------- ---------- ---------- ----------
EcabTPRX2      ---------- ---------- ---------- ---------- ---------- ---------- ---------- ---------- ---------- ----------
HsapTPRX1      GATCCCAGGC CCAATCTCAG GCCCATTCTC AGGCCCAAAC CCAGGCCCGA TCCCAGGCCC A---AACCCA GGCCCGATCC CAGGCCCAAT CTCAGGCCCG
HsapTPRX2      ---------- ---------- ---------- ---------- ---------- ---------- ---------- ---------- ---------- ----------
LcanTPRX1      ---------- ---------- ---------- ---------- ---------- ---------- ---------- ---------- ---------- ----------
MermTPRX1      ---------- ---------- ---------- ---------- ---------- ---------- ---------- ---------- ---------- ----------
MjavTPRX2      ---------- ---------- ---------- ---------- ---------- ---------- ---------- ---------- ---------- ----------
MmonTPRX1      ---------- ---------- ---------- ---------- ---------- ---------- ---------- ---------- ---------- ----------
MmonTPRX2      ---------- ---------- ---------- ---------- ---------- ---------- ---------- ---------- ---------- ----------
MmulTPRX1      GATCCCAGGC CCACTCGCTG GTCCACTCCC AGGCCCAATT TCAGGCCCA- ---------- ---------- ---------- ---------- ----------
MmulTPRX2      ---------- ---------- ---------- ---------- ---------- ---------- ---------- ---------- ---------- ----------
MmusCrxos-sv2  ---------- ---------- ---------- ---------- ---------- ---------- ---------- ---------- ---------- ----------
MmusCrxos-tv3  ---------- ---------- ---------- ---------- ---------- ---------- ---------- ---------- ---------- ----------
MmyoTPRX2a     ---------- ---------- ---------- ---------- ---------- ---------- ---------- ---------- ---------- ----------
MmyoTPRX2b     ---------- ---------- ---------- ---------- ---------- ---------- ---------- ---------- ---------- ----------
MmyoTPRX2c     ---------- ---------- ---------- ---------- ---------- ---------- ---------- ---------- ---------- ----------
MmyoTPRX2d     ---------- ---------- ---------- ---------- ---------- ---------- ---------- ---------- ---------- ----------
MmyoTPRX2e     ---------- ---------- ---------- ---------- ---------- ---------- ---------- ---------- ---------- ----------
MmyoTPRX2f     ---------- ---------- ---------- ---------- ---------- ---------- ---------- ---------- ---------- ----------
NleuTPRX1      GATCCCAGGT CCAATCCCAG GCCCAAACCC AGGCCTGATC CCAGGCCCAT TCCAGGCCCA GCCAGATCCC AGGCCCATTC CGTGGCCCAA T------CCC
NleuTPRX2      ---------- ---------- ---------- ---------- ---------- ---------- ---------- ---------- ---------- ----------
OariTPRX1      ---------- ---------- ---------- ---------- ---------- ---------- ---------- ---------- ---------- ----------
OariTPRX2      ---------- ---------- ---------- ---------- ---------- ---------- ---------- ---------- ---------- ----------
OcunTPRX2      ---------- ---------- ---------- ---------- ---------- ---------- ---------- ---------- ---------- ----------
PabeTPRX1      ---------- ---------- ---------- ---------- ---GGCCCA- ---------- ---------- ---------- ---------- ----------
PabeTPRX2      ---------- ---------- ---------- ---------- ---------- ---------- ---------- ---------- ---------- ----------
PdisTPRX1      ---------- ---------- ---------- ---------- ---------- ---------- ---------- ---------- ---------- ----------
PdisTPRX2      ---------- ---------- ---------- ---------- ---------- ---------- ---------- ---------- ---------- ----------
PleuCrxosA     ---------- ---------- ---------- ---------- ---------- ---------- ---------- ---------- ---------- ----------
PsinTPRX1      ---------- ---------- ---------- ---------- ---------- ---------- ---------- ---------- ---------- ----------
PsinTPRX2      ---------- ---------- ---------- ---------- ---------- ---------- ---------- ---------- ---------- ----------
RferTPRX1      ---------- ---------- ---------- ---------- ---------- ---------- ---------- ---------- ---------- ----------
RferTPRX2      ---------- ---------- ---------- ---------- ---------- ---------- ---------- ---------- ---------- ----------
SscrTPRX1      ---------- ---------- ---------- ---------- ---------- ---------- ---------- ---------- ---------- ----------
SscrTPRX2      ---------- ---------- ---------- ---------- ---------- ---------- ---------- ---------- ---------- ----------
SvulTPRX2      ---------- ---------- ---------- ---------- ---------- ---------- ---------- ---------- ---------- ----------
TbcTPRX2       ---------- ---------- ---------- ---------- ---------- ---------- ---------- ---------- ---------- ----------
UameTPRX1      ---------- ---------- ---------- ---------- ---------- ---------- ---------- ---------- ---------- ----------
ZcalTPRX1      ---------- ---------- ---------- ---------- ---------- ---------- ---------- ---------- ---------- ----------
ZcalTPRX2      ---------- ---------- ---------- ---------- ---------- ---------- ---------- ---------- ---------- ----------
```

```
               1501
BindTPRX1      ---------- ---------- ---------- ---------- ---------- ---------- ---------- ---------- ---------- ----------
BindTPRX2      ---------- ---------- ---------- ---------- ---------- ---------- ---------- ---------- ---------- ----------
BmusTPRX1      ---------- ---------- ---------- ---------- ---------- ---------- ---------- ---------- ---------- ----------
BmusTPRX2      ---------- ---------- ---------- ---------- ---------- ---------- ---------- ---------- ---------- ----------
BtauTPRX1      ---------- ---------- ---------- ---------- ---------- ---------- ---------- ---------- ---------- ----------
BtauTPRX2      ---------- ---------- ---------- ---------- ---------- ---------- ---------- ---------- ---------- ----------
CcanTPRX2      ---------- ---------- ---------- ---------- ---------- ---------- ---------- ---------- ---------- ----------
CcriTPRX1      ---------- ---------- ---------- ---------- ---------- ---------- ---------- ---------- ---------- ----------
CcriTPRX2      ---------- ---------- ---------- ---------- ---------- ---------- ---------- ---------- ---------- ----------
CferTPRX1      ---------- ---------- ---------- ---------- ---------- ---------- ---------- ---------- ---------- ----------
CferTPRX2      ---------- ---------- ---------- ---------- ---------- ---------- ---------- ---------- ---------- ----------
ClfTPRX1       ---------- ---------- ---------- ---------- ---------- ---------- ---------- ---------- ---------- ----------
ClfTPRX2       ---------- ---------- ---------- ---------- ---------- ---------- ---------- ---------- ---------- ----------
EcabTPRX1      ---------- ---------- ---------- ---------- ---------- ---------- ---------- ---------- ---------- ----------
EcabTPRX2      ---------- ---------- ---------- ---------- ---------- ---------- ---------- ---------- ---------- ----------
HsapTPRX1      ATCCCAGGCC CAATCTCAGT CCCGATCCCA GGCCCGATCC CAGGCCCAAT CTCAGGCCCA ATCTCAGGCC CAAACCCAGG CCCGATCCCA GGCCCAATCC
HsapTPRX2      ---------- ---------- ---------- ---------- ---------- ---------- ---------- ---------- ---------- ----------
LcanTPRX1      ---------- ---------- ---------- ---------- ---------- ---------- ---------- ---------- ---------- ----------
MermTPRX1      ---------- ---------- ---------- ---------- ---------- ---------- ---------- ---------- ---------- ----------
MjavTPRX2      ---------- ---------- ---------- ---------- ---------- ---------- ---------- ---------- ---------- ----------
MmonTPRX1      ---------- ---------- ---------- ---------- ---------- ---------- ---------- ---------- ---------- ----------
MmonTPRX2      ---------- ---------- ---------- ---------- ---------- ---------- ---------- ---------- ---------- ----------
MmulTPRX1      ---------- ---------- ---------- ---------- ---------- ---------- ---------- ---------- ---------- GCCCAGATTC
MmulTPRX2      ---------- ---------- ---------- ---------- ---------- ---------- ---------- ---------- ---------- ----------
MmusCrxos-sv2  ---------- ---------- ---------- ---------- ---------- ---------- ---------- ---------- ---------- ----------
MmusCrxos-tv3  ---------- ---------- ---------- ---------- ---------- ---------- ---------- ---------- ---------- ----------
MmyoTPRX2a     ---------- ---------- ---------- ---------- ---------- ---------- ---------- ---------- ---------- ----------
MmyoTPRX2b     ---------- ---------- ---------- ---------- ---------- ---------- ---------- ---------- ---------- ----------
MmyoTPRX2c     ---------- ---------- ---------- ---------- ---------- ---------- ---------- ---------- ---------- ----------
MmyoTPRX2d     ---------- ---------- ---------- ---------- ---------- ---------- ---------- ---------- ---------- ----------
MmyoTPRX2e     ---------- ---------- ---------- ---------- ---------- ---------- ---------- ---------- ---------- ----------
MmyoTPRX2f     ---------- ---------- ---------- ---------- ---------- ---------- ---------- ---------- ---------- ----------
NleuTPRX1      AGGCCC---- --------AA TCCCAGGCCC AGCCCAGATC CCGGGCCCAA TCTCAGGCCC AATCTC---- --AAGCCCAA ACCCAGGCCC AGCCCAGATC
NleuTPRX2      ---------- ---------- ---------- ---------- ---------- ---------- ---------- ---------- ---------- ----------
OariTPRX1      ---------- ---------- ---------- ---------- ---------- ---------- ---------- ---------- ---------- ----------
OariTPRX2      ---------- ---------- ---------- ---------- ---------- ---------- ---------- ---------- ---------- ----------
OcunTPRX2      ---------- ---------- ---------- ---------- ---------- ---------- ---------- ---------- ---------- ----------
PabeTPRX1      ---------- ---------- ---------- ---------- ---------- ---------- ATCTCAGACC CAAACCCAGG CCTGATCCCA GGC-------
PabeTPRX2      ---------- ---------- ---------- ---------- ---------- ---------- ---------- ---------- ---------- ----------
PdisTPRX1      ---------- ---------- ---------- ---------- ---------- ---------- ---------- ---------- ---------- ----------
PdisTPRX2      ---------- ---------- ---------- ---------- ---------- ---------- ---------- ---------- ---------- ----------
PleuCrxosA     ---------- ---------- ---------- ---------- ---------- ---------- ---------- ---------- ---------- ----------
PsinTPRX1      ---------- ---------- ---------- ---------- ---------- ---------- ---------- ---------- ---------- ----------
PsinTPRX2      ---------- ---------- ---------- ---------- ---------- ---------- ---------- ---------- ---------- ----------
RferTPRX1      ---------- ---------- ---------- ---------- ---------- ---------- ---------- ---------- ---------- ----------
RferTPRX2      ---------- ---------- ---------- ---------- ---------- ---------- ---------- ---------- ---------- ----------
SscrTPRX1      ---------- ---------- ---------- ---------- ---------- ---------- ---------- ---------- ---------- ----------
SscrTPRX2      ---------- ---------- ---------- ---------- ---------- ---------- ---------- ---------- ---------- ----------
SvulTPRX2      ---------- ---------- ---------- ---------- ---------- ---------- ---------- ---------- ---------- ----------
TbcTPRX2       ---------- ---------- ---------- ---------- ---------- ---------- ---------- ---------- ---------- ----------
UameTPRX1      ---------- ---------- ---------- ---------- ---------- ---------- ---------- ---------- ---------- ----------
ZcalTPRX1      ---------- ---------- ---------- ---------- ---------- ---------- ---------- ---------- ---------- ----------
ZcalTPRX2      ---------- ---------- ---------- ---------- ---------- ---------- ---------- ---------- ---------- ----------
```

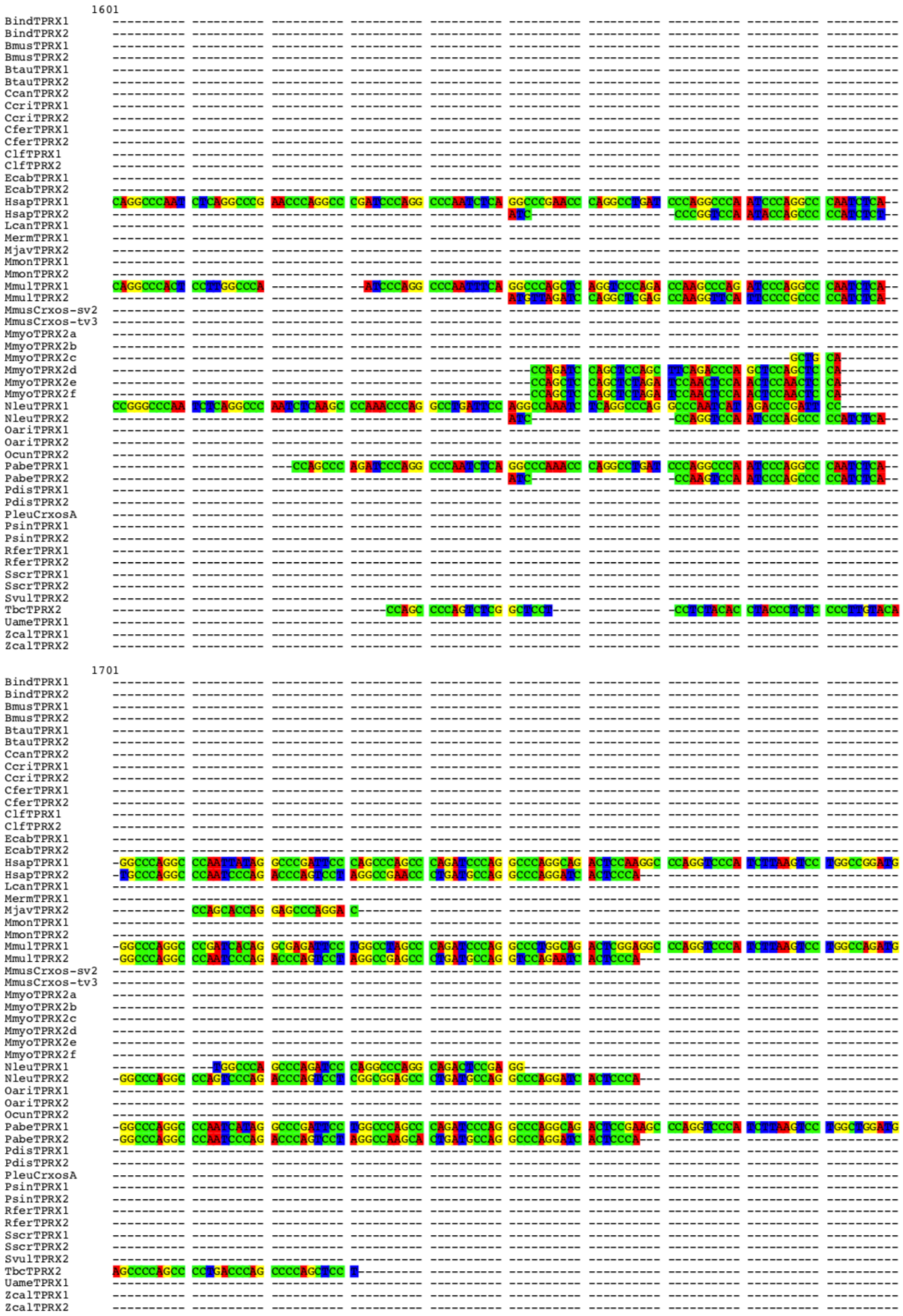
1601
1701

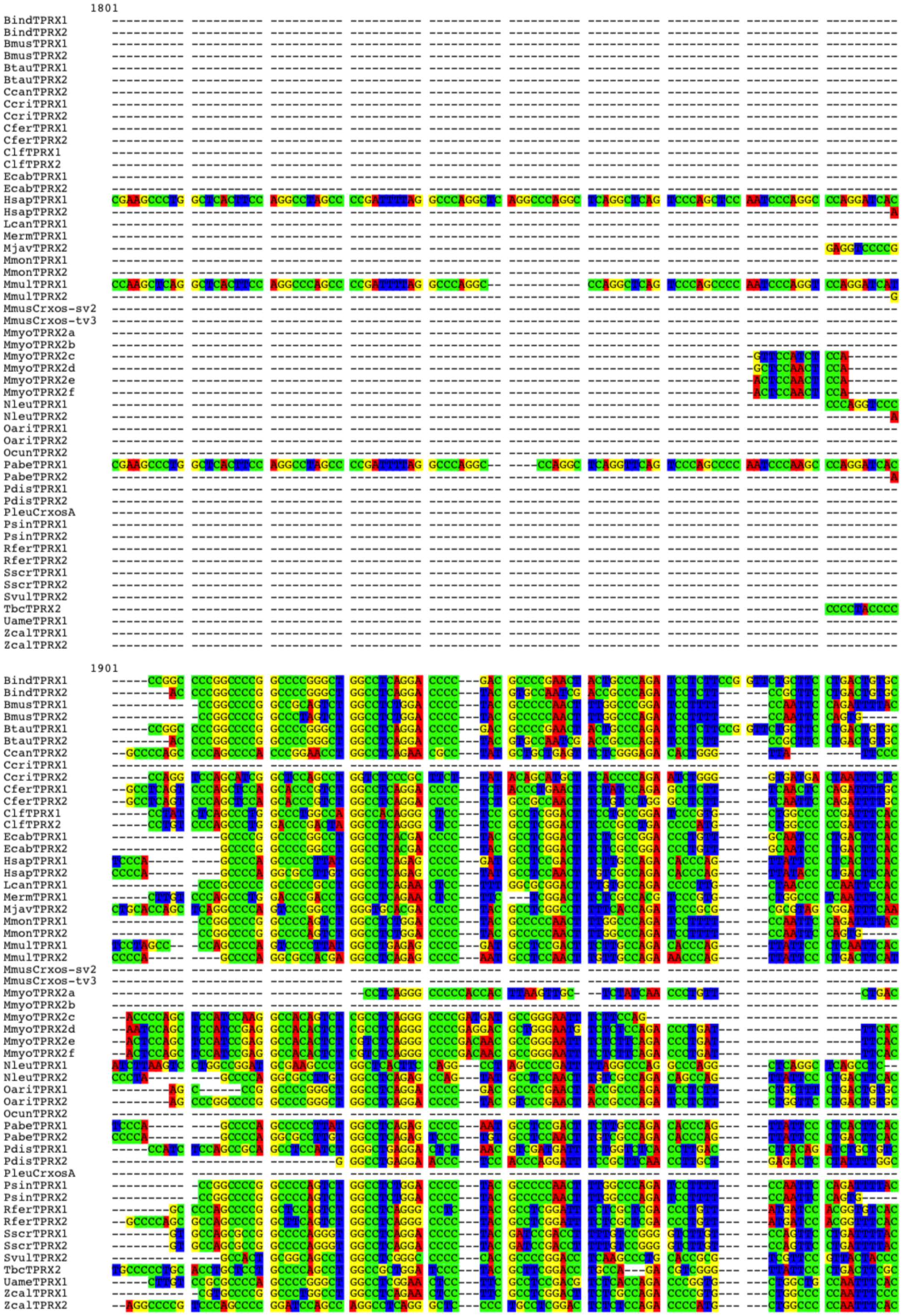

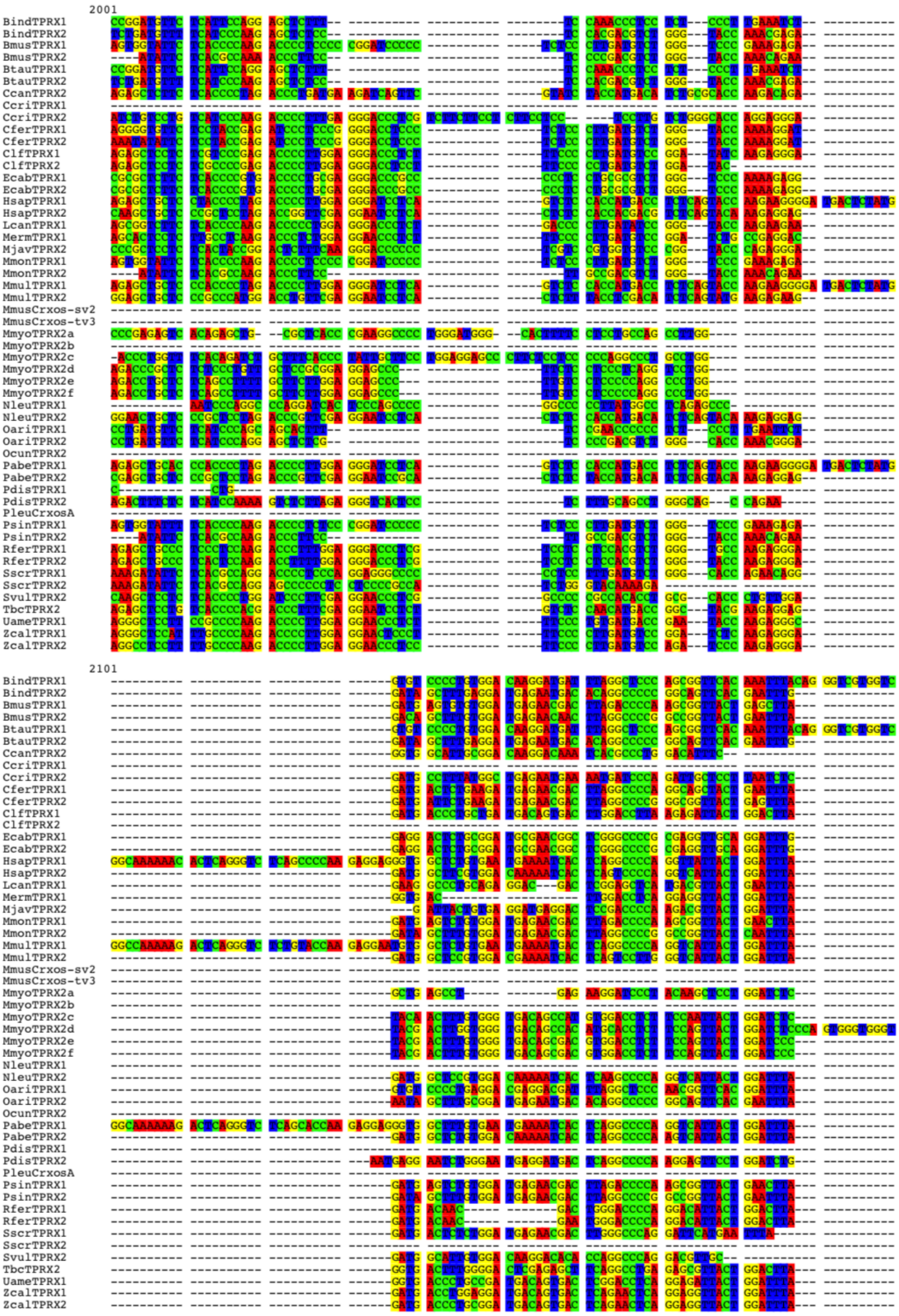
2001
BindTPRX1
BindTPRX2
BmusTPRX1
BmusTPRX2
BtauTPRX1
BtauTPRX2
CcanTPRX2
CcriTPRX1
CcriTPRX2
CferTPRX1
CferTPRX2
ClfTPRX1
ClfTPRX2
EcabTPRX1
EcabTPRX2
HsapTPRX1
HsapTPRX2
LcanTPRX1
MermTPRX1
MjavTPRX2
MmonTPRX1
MmonTPRX2
MmulTPRX1
MmulTPRX2
MmusCrxos-sv2
MmusCrxos-tv3
MmyoTPRX2a
MmyoTPRX2b
MmyoTPRX2c
MmyoTPRX2d
MmyoTPRX2e
MmyoTPRX2f
NleuTPRX1
NleuTPRX2
OariTPRX1
OariTPRX2
OcunTPRX2
PabeTPRX1
PabeTPRX2
PdisTPRX1
PdisTPRX2
PleuCrxosA
PsinTPRX1
PsinTPRX2
RferTPRX1
RferTPRX2
SscrTPRX1
SscrTPRX2
SvulTPRX2
TbcTPRX2
UameTPRX1
ZcalTPRX1
ZcalTPRX2
2101

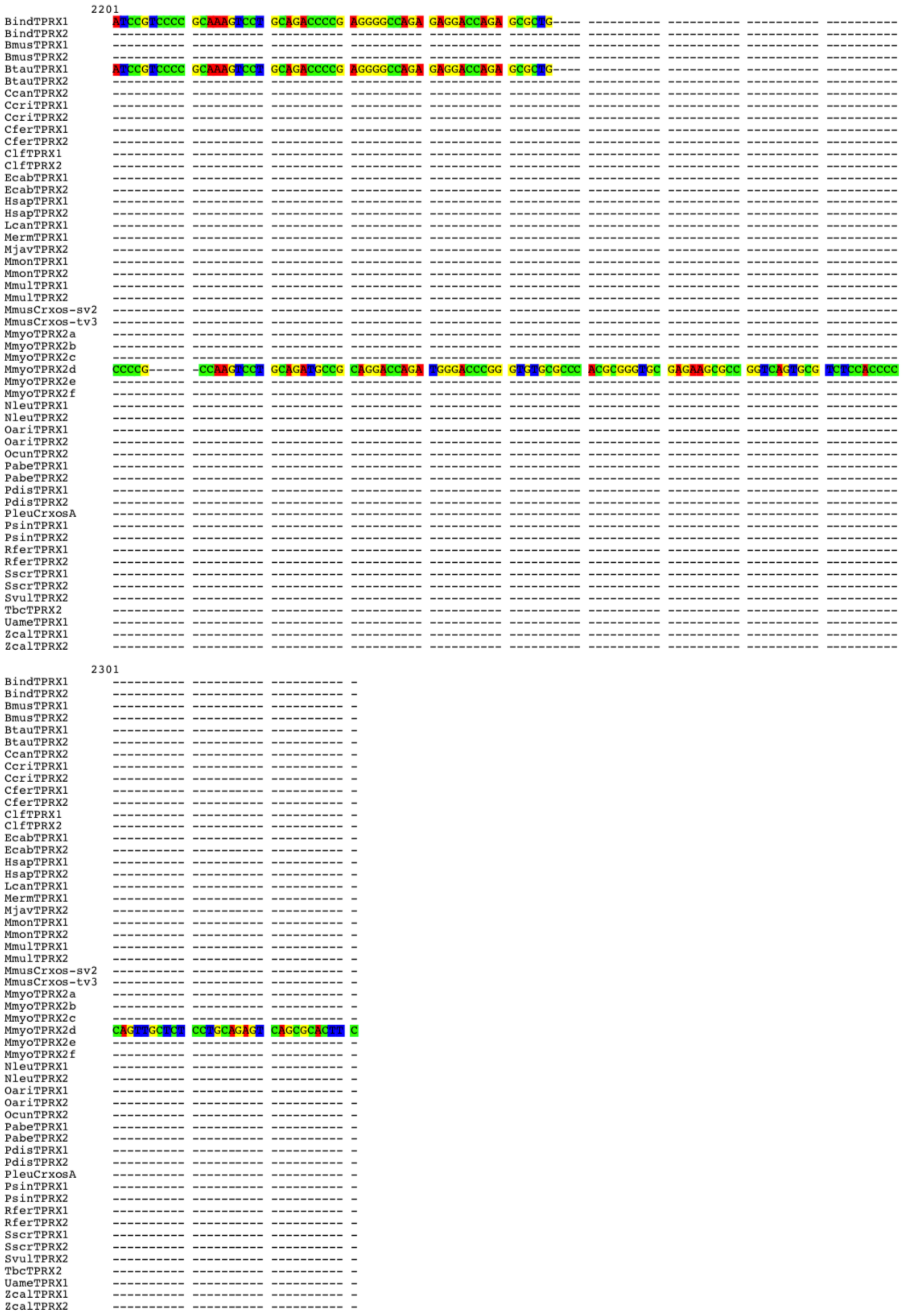

Online Resource – Fig. S5 Multiple sequence alignment of coding regions of *TPRX* genes used for gene conversion analysis. Stop codons were removed. Species abbreviations as in Fig. 2 of main text.

Online Resource – Fig. S6

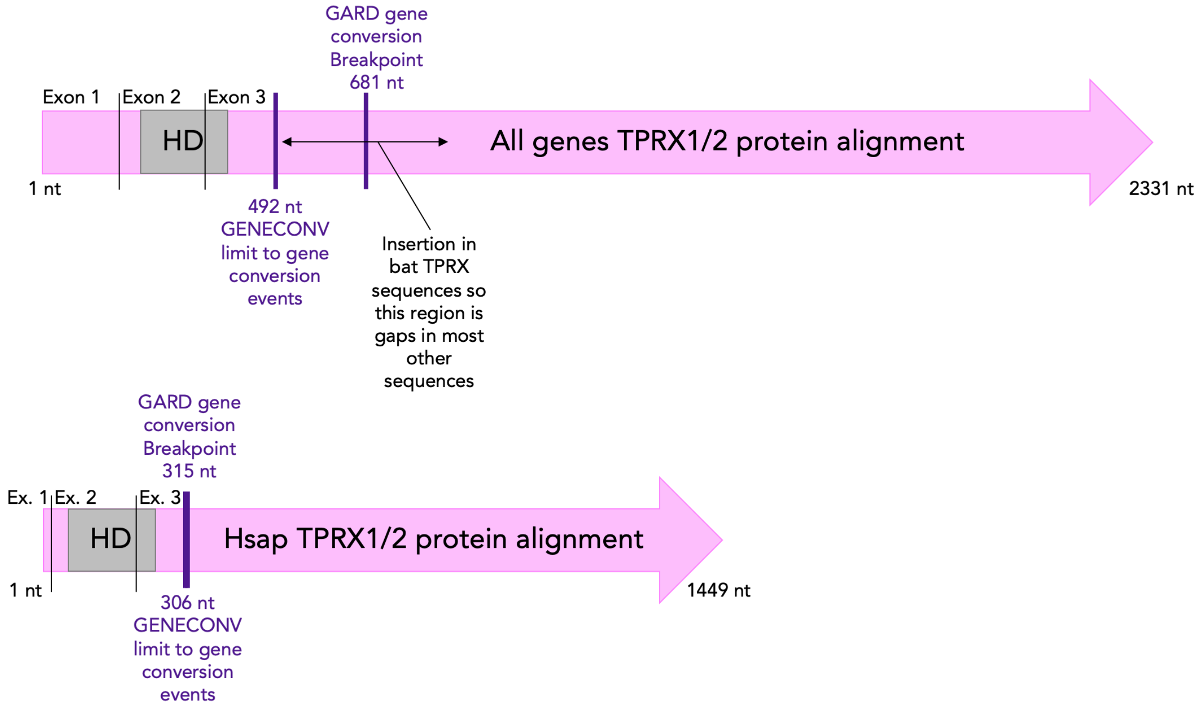

Online Resource – Fig. S6 Location of breakpoint identified by GARD (Kosakovsky Pond et al. 2006a, b) and limit to gene conversion identified by GENECONV (Sawyer 1989) in multiple sequence alignments of the *TPRX1* and *TPRX2* genes of all sampled species (top) and just *Homo sapiens* (bottom). GARD breakpoint and GENECONV gene conversion limit are 189 nucleotides apart in the 'all species' alignment primarily because of an insertion in bats. In most species they are much closer, and in humans the deduced breakpoints are only 9 nucleotides apart. Multiple sequence alignment of all *TPRX* genes is available as Online Resource – Fig. S5. Abbreviations: nt = nucleotide; Ex. = exon; HD = homeodomain, Hsap = *Homo sapiens*.

Online Resource – Fig. S7a

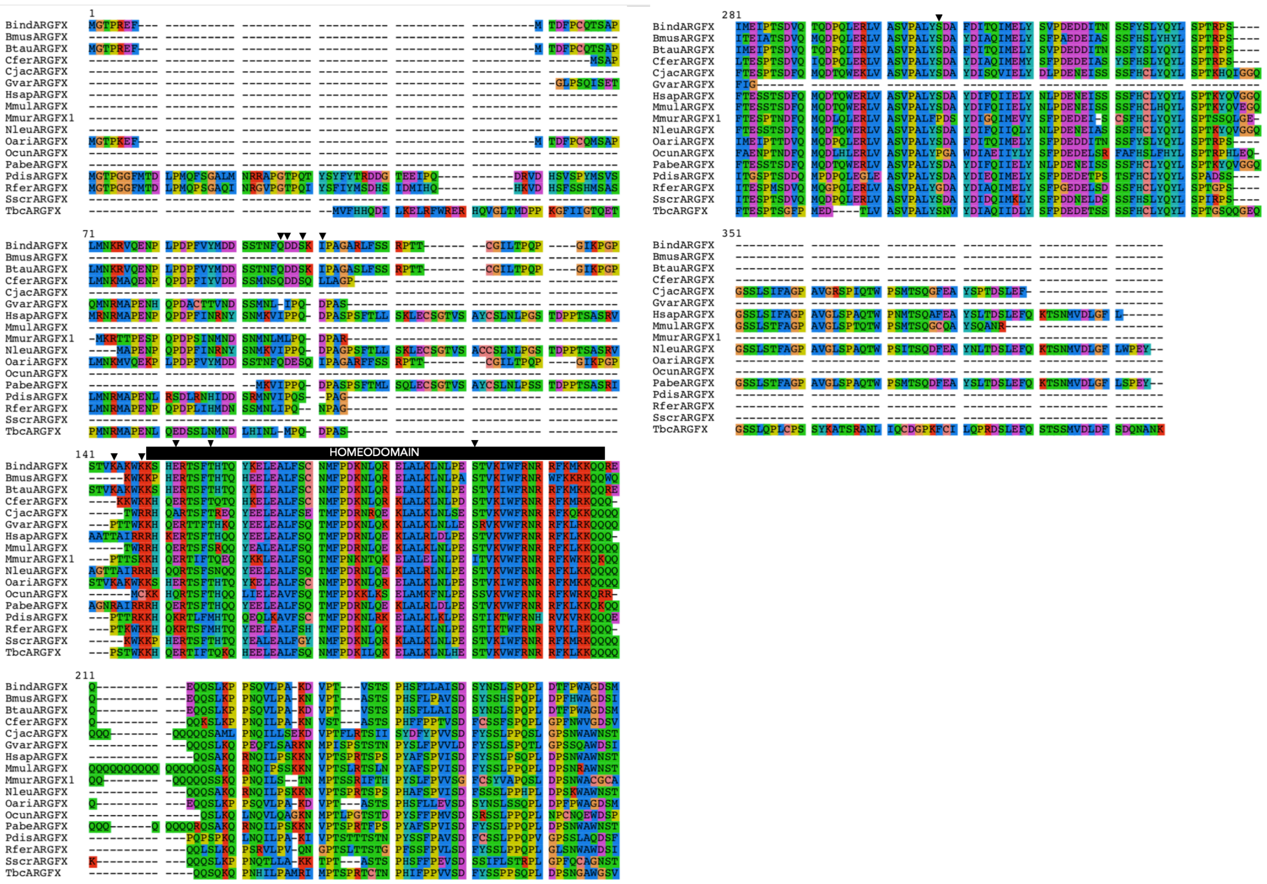

Online Resource – Fig. S7a Untrimmed multiple sequence alignment of ARGFX protein sequences. Sites under positive selection, as identified by MEME (Murrell et al. 2012), are marked with a black arrow. The homeodomain is marked with a black box. Species abbreviations as in Fig. 2 of main text.

Online Resource – Fig. S7b

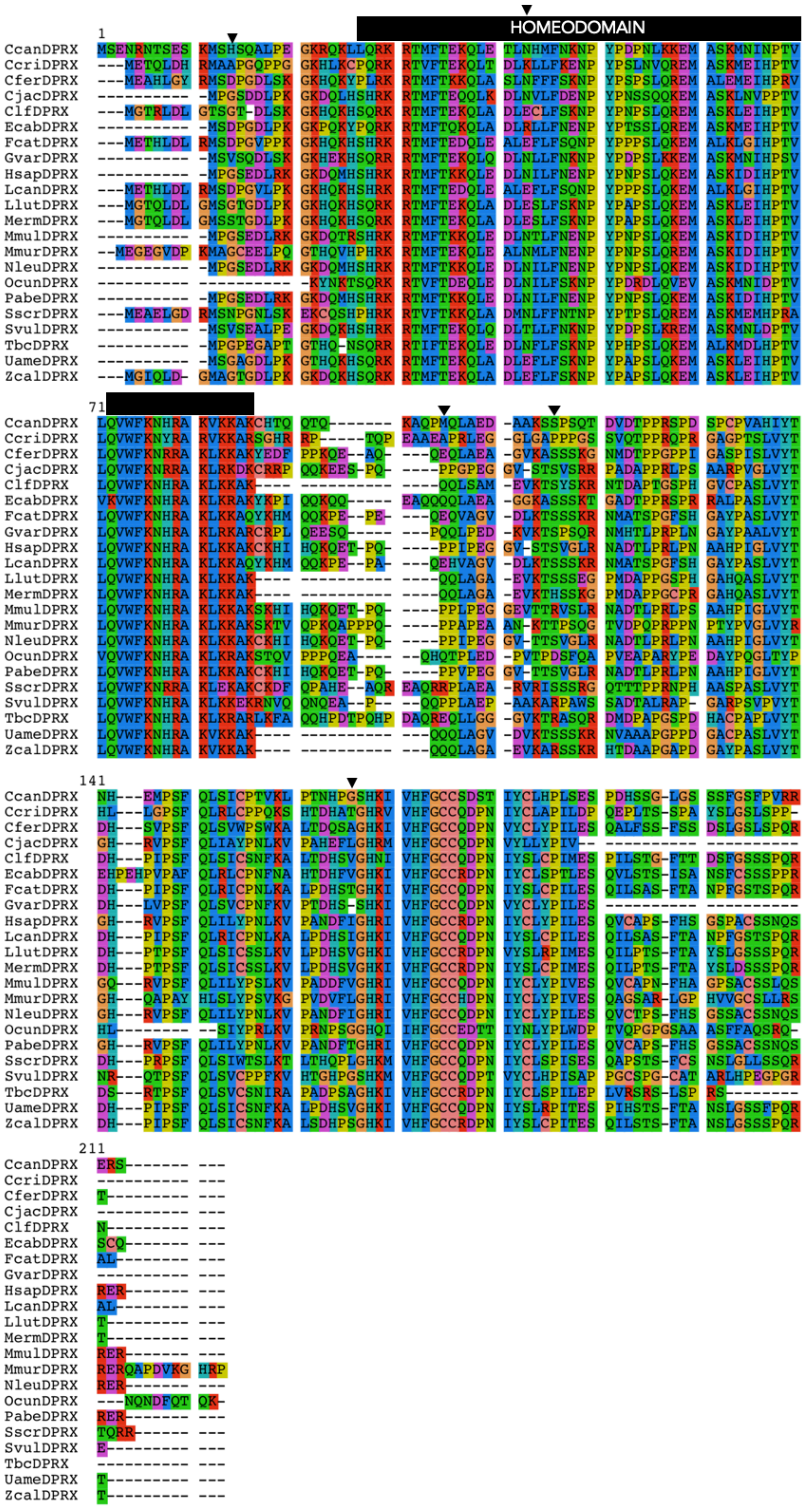

Online Resource – Fig. S7b Untrimmed multiple sequence alignment of DPRX protein sequences. Sites under positive selection, as identified by MEME (Murrell et al. 2012), are marked with a black arrow. The homeodomain is marked with a black box. Species abbreviations as in Fig. 2 of main text.

Online Resource – Fig. S7c

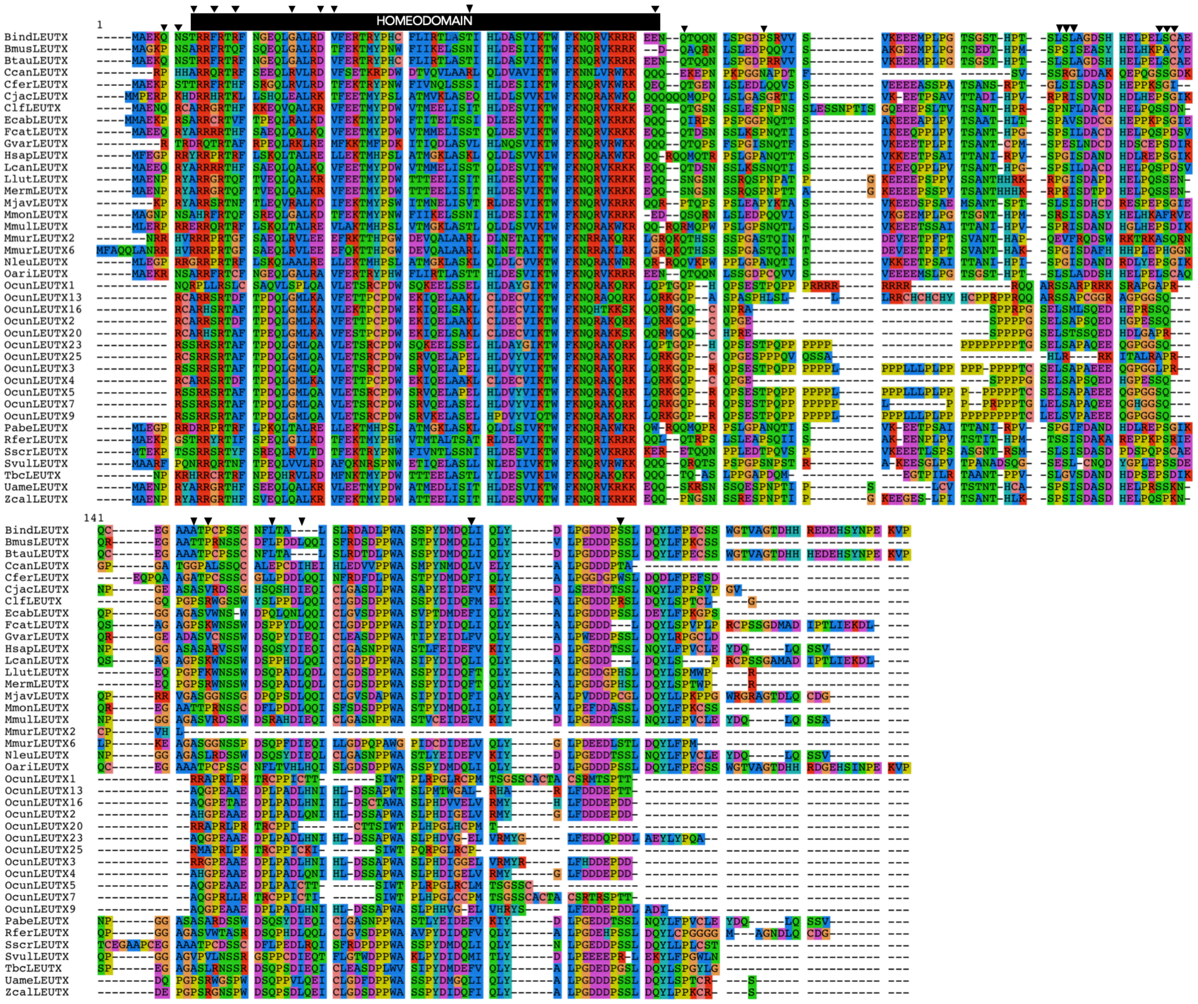

Online Resource – Fig. S7c Untrimmed multiple sequence alignment of LEUTX protein sequences. Sites under positive selection, as identified by MEME (Murrell et al. 2012), are marked with a black arrow. The homeodomain is marked with a black box. Species abbreviations as in Fig. 2 of main text.

Online Resource – Fig. S7d

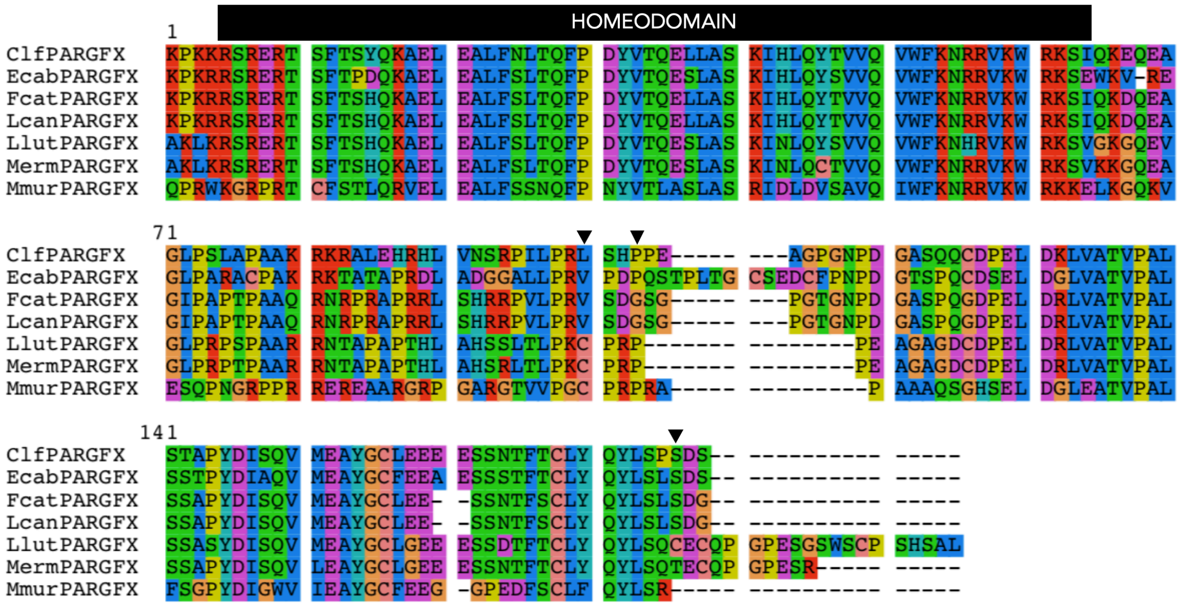

Online Resource – Fig. S7d Untrimmed multiple sequence alignment of PARGFX protein sequences. Sites under positive selection, as identified by MEME (Murrell et al. 2012), are marked with a black arrow. The homeodomain is marked with a black box. Species abbreviations as in Fig. 2 of main text.

Online Resource – Fig. S7e

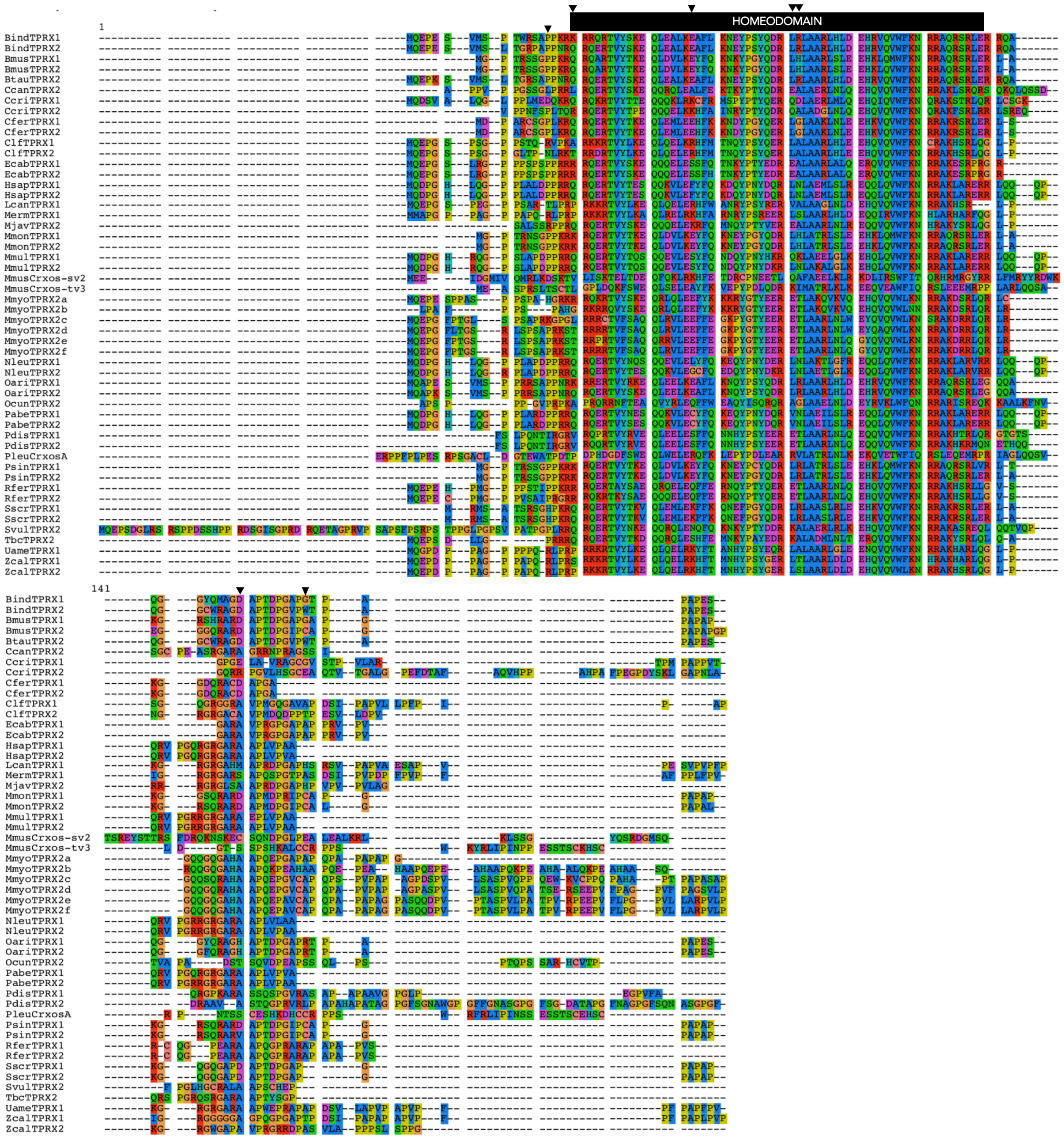

Online Resource – Fig. S7e Untrimmed multiple sequence alignment of TPRX partition 1 protein sequences. Sites under positive selection, as identified by MEME (Murrell et al. 2012), are marked with a black arrow. The homeodomain is marked with a black box. Species abbreviations as in Fig. 2 of main text.

Online Resource – Fig. S7f

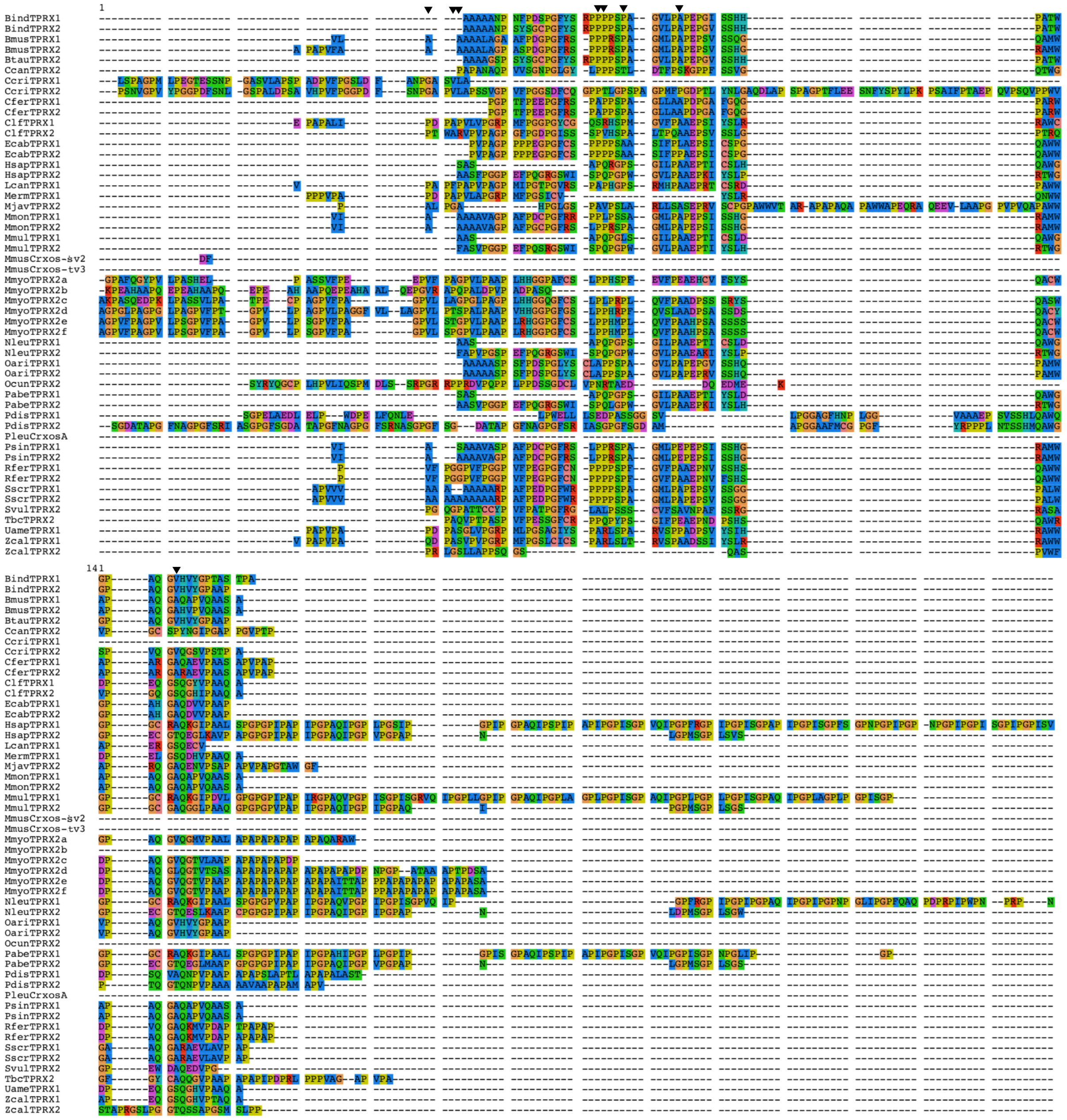

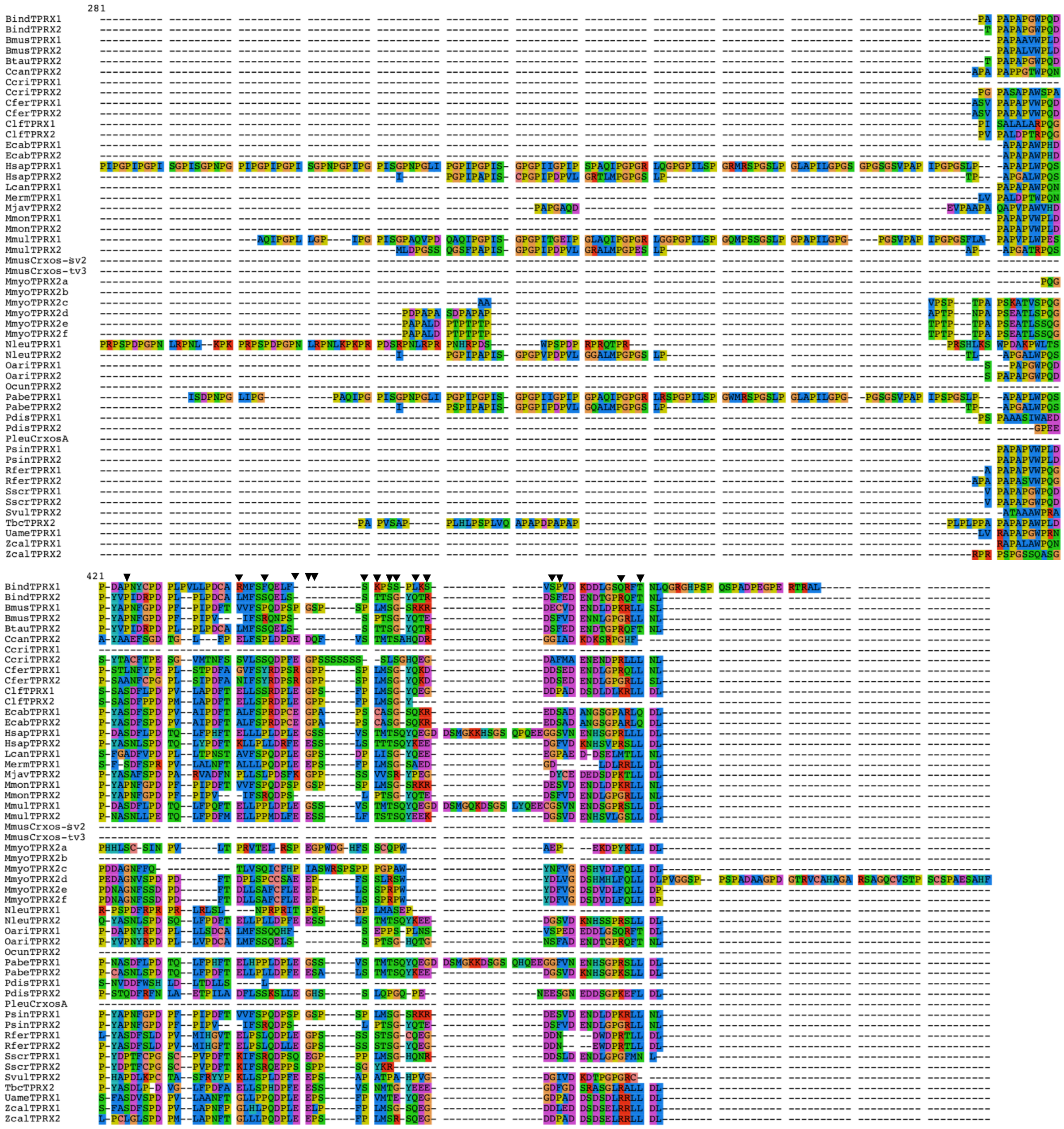

Online Resource – Fig. S7f Untrimmed multiple sequence alignment of TPRX partition 2 protein sequences. Sites under positive selection, as identified by MEME (Murrell et al. 2012), are marked with a black arrow. Species abbreviations as in Fig. 2 of main text.

Online Resource – Fig. S8

```
>HsapARGFX
    4    9                                        43
RHKERTSFTHQQYEELEALFSQTMFPDRNLQEKLALRLDLPESTVKVWFRNRRFKLKKQQ

>HsapDPRX
                17
SHRKRTMFTKKQLEDLNILFNENPYPNPSLQKEMASKIDIHPTVLQVWFKNHRAKLKKAK

>HsapLEUTX
1  4  7      14   18 19                 37                    60
YRRPRTRFLSKQLTALRELLEKTMHPSLATMGKLASKLQLDLSVVKIWFKNQRAKWKRQQ

>HsapTPRX1
1                18            32 33
QRQERTVYTESQQKVLEFYFQKDQYPNYDQRLNLAEMLSLREQQLQVWFKNRRAKLARER
```

Online Resource – Fig. S8 Homeodomains of *Homo sapiens* ETCHbox proteins with residues deduced to have been under positive selection highlighted in red. Numbers show the location of positively selected residues within the homeodomain.

Reference list

Chen K, Durand D, Farach-Colton M (2000) NOTUNG: A program for dating gene duplications and optimizing gene family trees. J Comput Biol. https://doi.org/10.1089/106652700750050871

De Bie T, Cristianini N, Demuth JP, Hahn MW (2006) CAFE: A computational tool for the study of gene family evolution. Bioinformatics. https://doi.org/10.1093/bioinformatics/btl097

Durand D, Halldórsson BV, Vernot B (2006) A hybrid micro-macroevolutionary approach to gene tree reconstruction. J Comput Biol. https://doi.org/10.1089/cmb.2006.13.320

Han M V., Thomas GWC, Lugo-Martinez J, Hahn MW (2013) Estimating gene gain and loss rates in the presence of error in genome assembly and annotation using CAFE 3. Mol Biol Evol. https://doi.org/10.1093/molbev/mst100

Kosakovsky Pond SL, Posada D, Gravenor MB, et al (2006a) GARD: A genetic algorithm for recombination detection. Bioinformatics. https://doi.org/10.1093/bioinformatics/btl474

Kosakovsky Pond SL, Posada D, Gravenor MB, et al (2006b) Automated phylogenetic detection of recombination using a genetic algorithm. Mol Biol Evol. https://doi.org/10.1093/molbev/msl051

Kumar S, Stecher G, Suleski M, Hedges SB (2017) TimeTree: A Resource for Timelines, Timetrees, and Divergence Times. Mol Biol Evol. https://doi.org/10.1093/molbev/msx116

Murrell B, Wertheim JO, Moola S, et al (2012) Detecting individual sites subject to episodic diversifying selection. PLoS Genet. https://doi.org/10.1371/journal.pgen.1002764

Sawyer S (1989) Statistical tests for detecting gene conversion. Mol Biol Evol. https://doi.org/10.1093/oxfordjournals.molbev.a040567
